# Fungus-originated genes in the genomes of cereal and pasture grasses acquired through ancient lateral transfer

**DOI:** 10.1038/s41598-020-76478-4

**Published:** 2020-11-16

**Authors:** Hiroshi Shinozuka, Maiko Shinozuka, Ellen M. de Vries, Timothy I. Sawbridge, German C. Spangenberg, Benjamin G. Cocks

**Affiliations:** 1 Centre for AgriBioscience, Agriculture Victoria, AgriBio, Bundoora, VIC 3086 Australia; 2 School of Applied Systems Biology, La Trobe University, Bundoora, VIC 3086 Australia

**Keywords:** Molecular evolution, Plant genetics, Transgenic plants

## Abstract

Evidence for ancestral gene transfer between *Epichloë* fungal endophyte ancestors and their host grass species is described. From genomes of cool-season grasses (the Poeae tribe), two *Epichloë*-originated genes were identified through DNA sequence similarity analysis. The two genes showed 96% and 85% DNA sequence identities between the corresponding *Epichloë* genes. One of the genes was specific to the Loliinae sub-tribe. The other gene was more widely conserved in the Poeae and Triticeae tribes, including wheat (*Triticum aestivum* L.) and barley (*Hordeum vulgare* L.). The genes were independently transferred during the last 39 million years. The transferred genes were expressed in plant tissues, presumably retaining molecular functions. Multiple gene transfer events between the specific plant and fungal lineages are unique. A range of cereal crops is included in the Poeae and Triticeae tribes, and the Loliinae sub-tribe is consisted of economically important pasture and forage crops. Identification and characterisation of the 'natural' adaptation transgenes in the genomes of cereals, and pasture and forage grasses, that worldwide underpin the production of major foods, such as bread, meat, and milk, may change the ‘unnatural’ perception status of transgenic and gene-edited plants.

## Introduction

The term genetically modified (GM) organism represents an organism ‘*in which the genetic material has been altered in a way that does not occur naturally by mating and/or natural recombination*’^1^. Plant individuals developed through use of transgenic techniques have, therefore, been regarded as GM organisms, partly due to absence of clear evidence for natural occurrence of such genetic modification^2,3^. The consequent stringent regulation has been a hurdle for commercialisation of transgenic technology-derived products, and equivalent restrictions are likely to be imposed on emerging gene-edited crops, particularly those with transgenes, despite the presence of scientific evidence indicating that potential risks of such GM products on human health and ecology do not substantially differ from those of non-GM counterparts^2–5^.

Horizontal gene transfer (HGT) is a known evolutionary mechanism in both prokaryotes and eukaryotes, permitting acquisition of adaptation novelty from taxonomically distant species^6^. Such events in multi-cellar eukaryotes are, however, less frequently documented, and identification of novel candidates from eukaryote genomes is required for understanding molecular basis of the mechanism, and the resulting effect on evolution in eukaryote lineages^7^. Angiosperms (flowering plants) represent multi-cellar plant spices, which relatively recently diverged [around 200 million years ago (MYA)] from other land plant species in the evolutionary time^8^. Following to the appearance, diversification of the lineages occurred during the last 100–125 million years, and the taxa is currently composed of around 300,000 spices, dominating both natural and cultivated landscapes^9^. Due to the importance as primary food source for human and animals, angiosperms have been major targets for genetics and genomics, especially for the crop breeding purposes^10^. The recent advances in DNA sequencing and computational technologies facilitated whole genome and transcriptome sequencing of angiosperm species, despite their expanded genome sizes, typically from a couple hundred mega to over ten giga base pairs in length, and complexity of transcriptome in them^11,12^.

Investigation of horizontally transferred genes has been commonly performed through a comparison of DNA sequences from taxonomically distant species, and a subsequent phylogenetic analysis, for identification of DNA sequences with an unusually high identity between the two distant lineages^13^. In plants, mitochondrial genes (sequences) transferred from other plants have been relatively frequently reported^14^. The genus *Amborella* represents a basal lineage of angiosperms, and, in amborella (*Amborella trichopoda* Baill.), extensive ancestral horizontal transfer of mitochondrial DNA genes was inferred^15^. The amborella mitochondrial genome was completely sequenced, to confirm the presence of an unusually large number of genes transferred from green alga, moss and angiosperm mitochondria. Due to the scale of integration, a mitochondria fusion-based model was proposed for the extensive HGT case^16^. Another well-characterised example in flowering plants is the genes transferred through *Agrobacterium*-based conjugation. The *Agrobacterium*-based event was originally identified in tree tobacco (*Nicotiana glauca* Graham, Solanaceae family)^17^. From the tree tobacco genome, a sequence showing a similarity to the *Agrobacterium* Ri plasmid was found, and multiple gene(-like) sequences were located on the *Agrobacterium*-derived insertion. Similar *Agrobacterium*-derived sequences have been identified from 15 *Nicotiana* species, suggesting multiple HTG events from *Agrobacterium* during the recent evolutionary time^18^. *Agrobacterium*-originated genes have also been identified in common toadflax (*Linaria vulgaris*, Plantaginaceae family) and sweet potato (*Ipomoea batatas* (L.) Lam, Convolvulaceae family)^19,20^. Recent studies have revealed potentially frequent nuclear gene exchanges between angiosperms, especially between host and parasitic species^21^. From purple witchweed (*Striga hermonthica* (Delile) Benth., Eudicots clade), which may infest plants in the Monocot clade, the *ShContig9483* gene was identified, showing an unusually high DNA sequence similarity to sorghum (*Sorghum bicolor* L.) and rice (*Oryza sativa* L.) genes. At the 3′ end of the *ShContig9483* sequence, consecutive adenine (A) nucleotides were found, suggesting a possibility of mRNA-mediated HGT from a Monocot species^22^. Through a systematic analysis for parasitic plants of the Orobanchaceae family, a total of 42 genes were identified as HGT candidates between parasitic and host plants, in addition to previously reported candidates^21^. Due to conservation of the intron positions between putative decedents of gene donor and recipient, a proportion of the candidates were suggested to have been integrated through a transformation-like mechanism. Among the 42 HGT candidates, 9 sequences were annotated as plant defence-related genes, suggesting that those genes may have contributed to pathogen resistance and/or parasite-host interaction.

No nuclear gene transferred from non-plant species into angiosperms has, however, been reported until recently^23^. In a previous systematic study for genomes of representative angiosperms [*Arabidopsis thaliana* (L.), rice, sorghum and poplar (*Populus trichocarpa* Torr. & A.Gray ex. Hook.)], no evidence for an HGT event from the fungal linage was found, in contrast to identification of two and three candidates in moss and lycophyte lineages, respectively^13^. It had, therefore, been believed that such an event hardly or never happened in the angiosperm lineage. The β-1,6-glucanase-like gene (*Lp*BGNL) of perennial ryegrass (*Lolium perenne* L.; Poeae tribe, Loliinae sub-tribes) was the first documented candidate for an ancestral nuclear gene transfer event from non-plant species into the angiosperm lineage. A high sequence similarity (90%) to the corresponding gene of *Epichloë* species inferred that the gene was transferred from an ancestral species of the *Epichloë* taxon, which composes a sister group to the ergot fungi (the genus *Claviceps*)^24^. As asexual *Epichloë* species may establish symbiotic relationships between the Loliinae and Dactylidinae species, close physical contact may have facilitated the HGT. More recently, a glutathione S-transferase gene (*Fhb7*) was identified from a distant relative of wheat, *Thinopyrum elongatum*^25,26^. This gene showed up to 97% DNA sequence similarity to the corresponding genes in *Epichloë* species, suggesting another transfer event from the *Epichloë* lineage. Due to contribution to fungal-pathogen resistance, especially against *Fusarium*, the *Fhb7* gene is expected to be valuable for wheat breeding.

In the current report, identification of additional HGT candidates in cool-season grass species is described. The genes are likely to have been transferred from the *Epichloë* lineage, and the identification of the non-plant species-originated genes in fully-sequenced crop species evidences that modern biotechnology-like genetic modification has naturally happened in food crop species. Characterisation of such 'natural' adaptation transgenes may increase public acceptance of advanced genetic improvement technologies.

## Results

An Illumina short-read sequencing library was prepared using short RNA from an endophyte-absent (E^−^) perennial ryegrass individual. To catalogue expressed non-polyadenylated [poly(A)] RNA from perennial ryegrass, the sequencing library was analysed on the Illumina MiSeq platform. A single Illumina MiSeq run generated a total of 8,216,014 reads. Subsequently, a dataset of unique short RNA reads, including 1,424,274 sequences, was generated (Supplementary Information 1). To identify perennial ryegrass genes potentially derived from the fungal taxa, a BLAST search of the perennial ryegrass (E^−^) transcriptome shotgun assembly (TSA) and unique short RNA read datasets was performed against the *Epichloë festucae* transcriptome^23,27^. Totals of 88 and 121 sequence similarity hits were identified from the TSA and unique short RNA read datasets (Supplementary Information 2 and 3). A manual examination was performed using the NCBI BLAST function, to eliminate sequences derived from microbiome, those related to highly conserved genes between the two taxa, such as actin and ubiquitin genes, and low confident matches. The manual examination initially excluded 116 hits due to low sequence similarity. A total of 76 hits were related to highly conserved genes between eukaryotes, and 12 hits were concluded to be contribution from plant-related microbiome. After exclusion of those hits, 5 were found to be putative HGT-related hits, and those hits were corresponding to two *E. festucae* genes, and the β-1,6-glucanase gene (Supplementary Information 4). From the TSA data, a sequence [unique identifier (UI): ID_150936_C1449060_17.0] showing a relatively high similarity (85%) to an *E. festucae* gene (UI: EfM3.066060.partial-2.mRNA-1) was identified. As the predicted product of the *Epichloë* gene showed a sequence similarity to a fungal transcriptional regulatory (FTR) protein (Genbank UI: CRL18938; identity: 48%, e-value: 2e−150) of *Penicillium camemberti* (Ascomycota, Trichocomaceae), the perennial ryegrass gene was designed *Lp*FTRL (*L. perenne* FTR-like). The other candidate was identified from the non-polyadenylated RNA unique reads (Sequence UI: 734684-1), and designated *Lp*DUF3632, due to a similarity (96%) to the *E. festucae* DUF3632 (domain of unknown function 3632)-like gene (UI: EfM3.028800.mRNA-1). Sequence contigs containing *Lp*FTRL and *Lp*DUF3632 from shotgun whole genome sequencing assembly data of the perennial ryegrass Impact_04_ genotype (E^−^) were identified^23^. The contigs were deposited into the NCBI GenBank (UIs: MG680924 for *Lp*FTRL and MG680923 for *Lp*DUF3632), of which sizes were 17.2 kb and 6.1 kb in length, respectively. Although in the *E. festucae* (Strain: Fl1) genome contigs, flanking genes to the *FTRL* and *DUF3632* sequences were identified within distances of 1–2 kb and 3–5 kb, respectively, no such flanking gene was predicted from the perennial ryegrass contigs. Instead, plant repetitive element-like sequences were identified in both contigs. A putative intron was identified for *Lp*DUF3632, while no intron was predicted for *Lp*FTRL.

**Figure 1:**
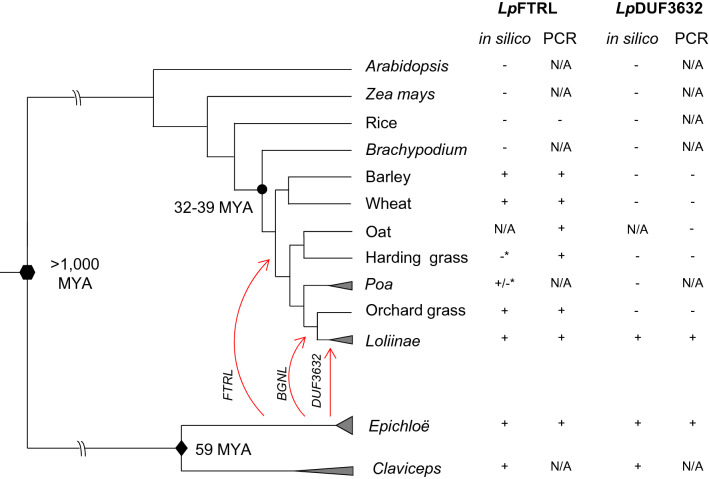
Presence/absence status of the *Lp*FTRL and *Lp*DUF3632-like sequences. The divergent point of plant species from other species (fungi and animals) is indicated with the filled hexagon (> 1,000 MYA), and the filled circle indicate the divergent point (32–39 MYA) of species belonging to the Triticeae and Poeae tribes from other Poaceae species. The filled diamond indicates the divergent point (59 MYA) of *Epichloë* species from *Claviceps* species. The triangles represent species belonging to the Loliinae or *Poa* subtribe, or *Epichloë* or *Claviceps* genera. The presence/absence status of putative homologues (orthologues) is shown on the right side of species/subtribe/genius name, in witch ‘In silico’ and ‘PCR’ denote the database and PCR-based screening results, respectively. The plus( +), minus( −), and ‘N/A’ denote ‘presence’, ‘absence’, and ‘not analysed’, respectively. Although results of database-based screening for *Lp*FTRL-like sequences in harding grass and one of *Poa* species were negative, indicated with an asterisk(*), the PCR-based assay indicated presence of orthologues in harding grass. Further details about database-based screening can be found in Supplementary Information 5. The putative periods of the ancient HGT events for *FTRL*, *DUF3632*, and *BGNL* are shown with red arrows.

A further in silico examination was performed to eliminate a possibility of DNA sequencing artifacts or contribution from microbiome. Through a BLASTN search against nucleotide sequences catalogued in the NCBI GenBank database, relatively high sequence similarity matches against *Lp*FTRL were identified from Tausch's goatgrass (*Aegilops tauschii* L., UI: XM_020322891.1) and barley (UI: AK375773.1), followed by ascomycota species, while only fungal genes showed a significant sequence similarity to *Lp*DUF3632 (Table 1). From publicly available barley and hexaploid wheat genome sequences, putative orthologues for *Lp*FTRL were identified in barley chromosomes 1H and 7H (Ensembl UI: HORVU1Hr1G009870 and HORVU7Hr1G108080, respectively), while the corresponding sequences were found from chromosome 7 of each sub-genome of hexaploid wheat (TraesCS7A02G564400, TraesCS7B02G375200, and TraesCS7D02G460600). Significant matches to *Lp*FTRL was also identified from the NCBI short read archive (SRA) data of cereal rye (*Secale cereal* L.) chromosome 7 (SRA UI: ERX140518). The *Lp*DUF3632 sequence was subjected to a BLASTN search against NCBI SRA data from cool-season grass species. Significant sequence matches were found from Italian ryegrass (*L. multiflorum* L.) and tall fescue (*Festuca arundinacea* L.), but not from orchard grass (*Dactylis glomerata* L.) or Antarctic hairgrass (*Deschampsia Antarctica* É.Desv.) (Fig. 1 and Supplementary Information 5). Significant matches to *Lp*FTRL were found from SRA data of cool-season grass species, except for transcriptome shotgun sequencing SRA of harding grass (*Phalaris aquatica* L.). As a control experiment, *Lp*BGNL, the perennial ryegrass architecture candidate genes, *Lp*ABCG5 and 6 (GenBank UIs: JN051254.1 and JN051255.1), and an *Epichloë*-specific gene, makes caterpillars floppy (mcf)-like gene (*EfMCF*, GenBank UI: KJ502561.1) were sought to find significantly matching sequences for *Lp*BGNL, and *Lp*ABCG5 and 6, but no sequence for the mcf-like gene from each SRA dataset^28,29^. Sequences corresponding to the two candidate genes were sought in genomes of other fully-sequenced angiosperm species, *Brachypodium* [*B. distachyon (L.) P. Beauv.*], rice, sorghum, *Zea Mays* (L.) and *A. thaliana*, and no significant match was, however, obtained. To confirm the gene presence/absence status, a PCR-based assay was performed. Presence of the *DUF3632*-like sequence in *Loliinae* species [perennial ryegrass, darnel (*L. temulentum* L.), tall fescue, and sheep fescue (*F. ovina* L.)] was confirmed, while no PCR amplification was observed from orchard grass and harding grass. Presence of *FTRL*-like sequence was confirmed in all tested Poeae species, including harding grass (Fig. 1, and Supplementary Information 6).

**Table 1:** Result of the DNA sequence similarity search for *Lp*FTRL and *Lp*DUF3632. ‘GPUK’ denotes for genome project at University of Kentucky. ‘N.A.’ stands for ‘not applicable’.

Candidates	Database	Sequence UI	Species	Kingdom	Max score	Total score	Query cover (%)	E value	Identity (%)
*Lp*FTRL	GPUK	scaffold00520	*Epichloë festucae*(strain:E2368)	Fungus	440	N.A	N.A	1.00E-122	86
	NCBI	XM_020322891.1	*Aegilops tauschii* Coss	Plant	1975	1975	84	0	87
		AK375773.1	*Hordeum vulgare* L	Plant	1925	2000	89	0	86
		XM_006963607.1	*Trichoderma reesei*	Fungus	563	563	67	5.00E−156	70
		XM_018802990.1	*Trichoderma gamsii*	Fungus	500	500	61	5.00 E−137	70
		XM_014089277.1	*Trichoderma atroviride*	Fungus	491	491	61	3.00 E−134	70
		XM_014098502.1	*Trichoderma virens*	Fungus	489	489	65	9.00 E−134	69
		XM_007788513.1	*Endocarpon pusillum* Hedw	Fungus	489	489	68	9.00 E−134	69
		XM_003049115.1	*Nectria haematococca*	Fungus	430	430	60	8.00 E−116	69
*Lp*DUF3632	GPUK	scaffold00067	*Epichloë festucae*(strain:E2368)	Fungus	525	N.A	N.A	1.00 E−148	88
	NCBI	XM_009222704.1	*Gaeumannomyces tritici*	Fungus	113	172	57	1.00 E−20	67
		XM_007285547.1	*Colletotrichum gloeosporioides*	Fungus	91.5	91.5	18	4.00 E−14	72

**Figure 2:**
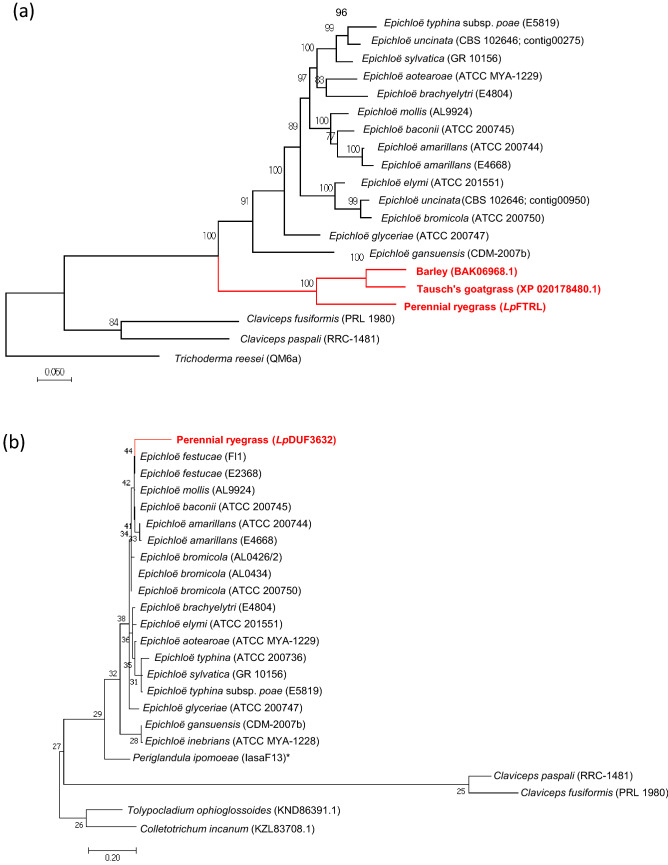
Phylogenetic tree of *Epichloë*-Triticeae/Poeae HGT candidates and corresponding fungal genes. The phylograms for *Lp*FTRL (**a**) and *Lp*DUF3632 (**b**) generated based on predicted amino acid sequences. Sequences from plant species are shown in red. For *Lp*FTRL, the sequence from *Trichoderma* was selected as an outer group to obtain a root of the phylogenic tree (**a**). For *Lp*DUF3632, amino acid sequences from *Tolypocladium* and *Colletotrichum* were selected as an outer group. A DUF3632-like gene was found from *Periglandula ipomoeae,* which is shown with an asterisk(*) (**b**). For fungus sequences, strain and sequence identifiers of the Genome Project at the University of Kentucky are shown in brackets. For plant sequences, NCBI GenBank UI is shown in brackets.

**Figure 3:**
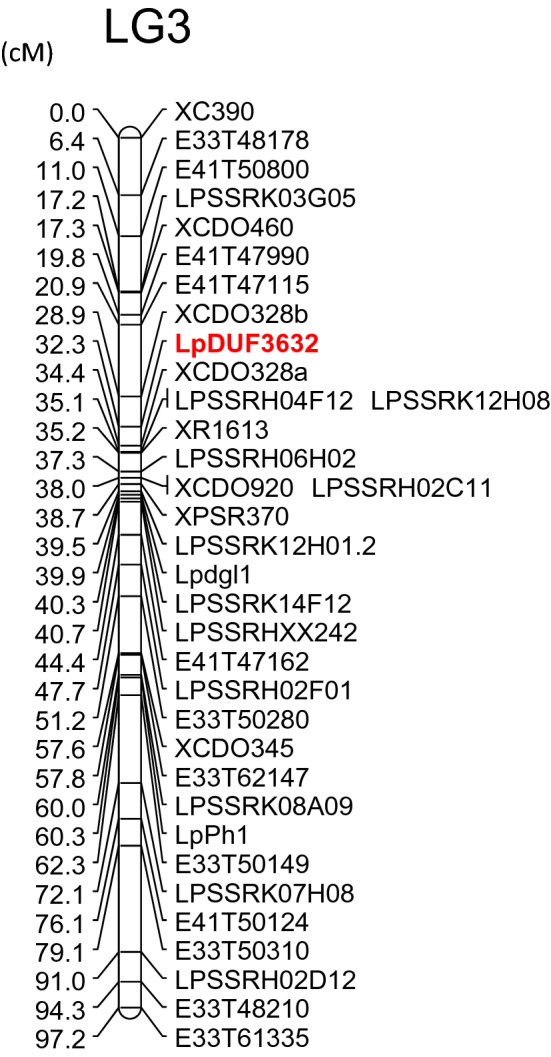
Genetic linkage mapping of *Lp*DUF3632. The *Lp*DUF3632-related DNA marker is shown in red. Genetic distance is shown on the left side of the linkage map.

A phylogenetic analysis was performed using the *FTRL* and *DUF3632* sequences from plant and fungal taxa. Close relationships of plant *FTRL* and *DUF3632*-like sequences between corresponding *Epichloë* sequences were revealed (Fig. 2). An *Lp*DUF3632-derived DNA-based marker was developed, and a genetic linkage analysis was performed, using the p150/112 reference linkage mapping population of perennial ryegrass (Supplementary Information 7). The marker was assigned into perennial ryegrass linkage group (LG) 3 (Fig. 3), which corresponds to the chromosome 3 of Triticeae species.

**Figure 4:**
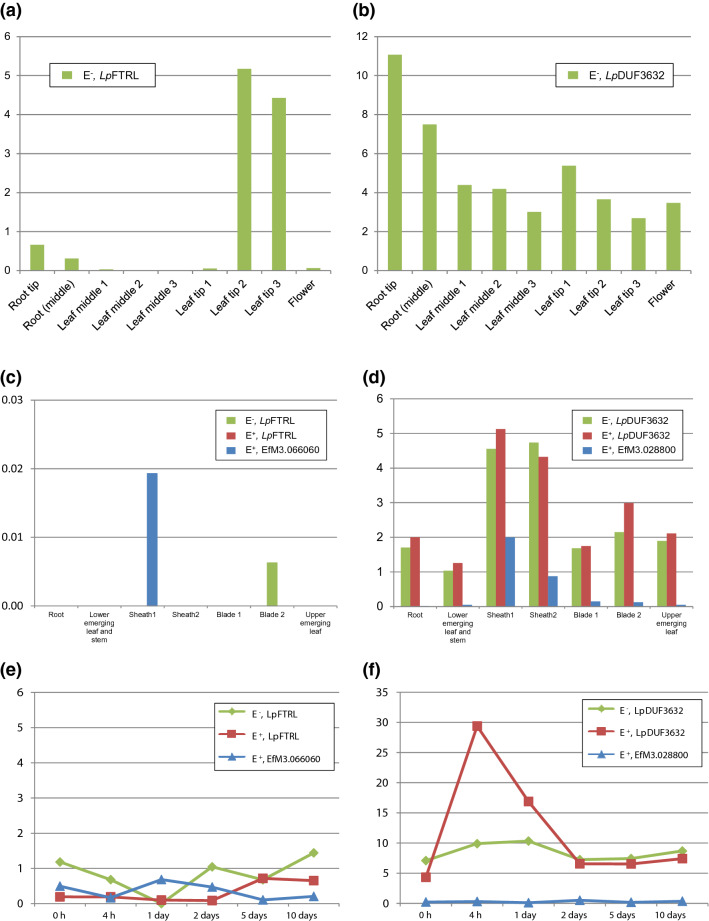
The expression levels of HGT candidates and corresponding fungal genes in perennial ryegrass. (**a**, **b**) The expression levels of *Lp*FTRL and *Lp*DUF3632 in each tissues of the perennial ryegrass Impact_04_ genotype. (**c**, **d**) The expression levels of the HGT candidates and corresponding fungal genes in E^+^ and E^−^ perennial ryegrass genotypes. (**e**, **f**) The time course gene expression analysis for the HGT candidates and corresponding fungal genes in perennial ryegrass seeds and young seedlings. The x-axis shows tissue types (**a**–**d**) or period after a germination treatment(**e**, **f**), and normalised read count numbers [counts per million (CPM)] are indicated on the y-axis. The green and red bars or lines show the expression levels of the HGT candidates in E^−^ and E^+^ plants, respectively, and the blue bars or lines show the expression levels of corresponding fungal genes in E^+^ plants. Note that the scales of the y-axes are not uniformed.

A gene expression analysis was performed, based on a read count approach of short read sequencing data. From the E^−^ perennial ryegrass transcriptome data^23^, a leaf tip-specific expression pattern of *Lp*FTRL was proposed (Fig. 4a and Supplementary Information 8). Although the counts per million reads (CPM) were relatively low compared with those of *Lp*BGNL, *Lp*DUF3632 was ubiquitously expressed (Fig. 4b). The SRA data from endophyte-infected (E^+^) and E^−^ perennial ryegrass plants, deposited by Massey University, were also subjected to the analysis. The SRA data did not include a leaf tip sample, and the expression level of *Lp*FTRL was below the limit of detection in most tested tissues, regardless of the E^+^/E^−^ status. Expression of the corresponding *Epichloë* gene (EfM3.066060) was not also observed in most tested tissues of the E^+^ plants (Fig. 4c and Supplementary Information 9). Expression of *Lp*DUF3632 was observed in all tested tissues, and the expression patterns were similar between E^+^ and E^−^ plants. Expression of the *Epichloë* DUF3632 gene (EfM3.028800) was detected from all tested tissues of the E^+^ plant, with a relatively high expression level in sheath (Fig. 4d). From short-read transcriptome sequencing data of the E^+^ and E^−^ perennial ryegrass seeds/young seedlings^23^, an increment of *Lp*DUF3632 expression level was observed in E^+^ seeds after a germination-treatment, although the expression levels of *Lp*FTRL, EfM3.066060, and EfM3.028800 stayed low throughout the tested 10 days (Fig. 4e,f, and Supplementary Information 10).

**Figure 5:**
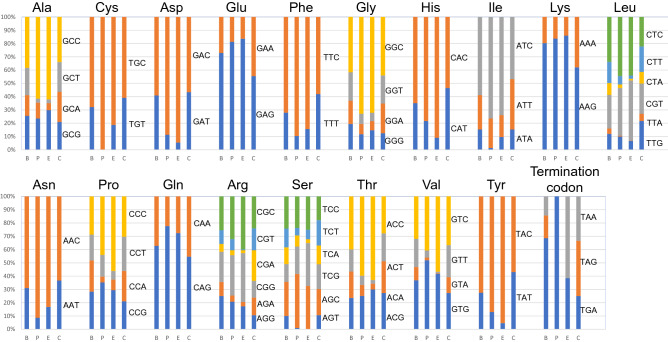
Visualised codon usage ratio for 18 amino acids and the termination signal. The y-axis shows the ratio, and ‘B’, ‘P’, ‘E’, and ‘C’ on the x-axis denote for codon usages in ‘barley (*Hordeum vulgare* subsp. *vulgare*) 1,490 coding sequences (CDSs)’, ‘plant *FTRL* genes’, ‘*Epichloë* FTRL genes’, and ‘*Claviceps purpurea* 37 CDSs’, respectively. Each color (dark blue, orange, grey, yellow, light blue or green) represents the ratio of each codon, of which DNA sequence is shown on the right side of data from the *C. purpurea* 37 CDSs. The usage information of barley and *C. purpurea* CDSs was obtained from the Codon Usage Database (https://www.kazusa.or.jp/codon/), and shown in the figure as codon usage reference in plant and fungal species.

Nucleotide substitution ratio was calculated for the horizontally transferred genes. The synonymous substitution rate (Ks/million years) of the plant *FTRL* sequences was not substantially different from those from fungi sequences (Table 2). Although some variation was observed in the non-synonymous substitution rates (Ka/million years), the rate from the plant sequences was between those from the *Claviceps-Epichloë* and *E. gansuensis*-other *Epichloë* combinations. The Ka/Ks ratio for the plant *FTRL* sequences were 0.376, which was close to that of the *E. gansuensis*-other *Epichloë* combination. A comparison with *E. festucae* DUF3632 indicated a similar Ka/Ks ratio (0.31) for *Lp*DUF3632 (Supplementary Information 11). Comparison of codon usage of *Lp*FTRL orthologues from diploid plant species and corresponding *Epichloë* sequences revealed a similar usage ratio for each amino acid, except for the termination codons (Fig. 5). Only ‘TGA’ was used in the plant genes as termination codon, the ratio of ‘TGA’ was only 38.5% in the *Epichloë* sequences.

**Table 2:** Ka and Ks rates for *Lp*FTRL orthologues and corresponding *Epichloë* and *Claviceps* genes. The ratios calculated from the *Claviceps* and *Epichloë*, *Claviceps* and Plants, Triticeae and Poeae (plants), and *E. gansuensis* and the rest of *Epichloë* combinations are shown as *Claviceps*-*Epichloë*, *Claviceps*-Plants, Triticeae—Poeae, and *E. gansuensis*-other *Epichloë*, respectively. As *Epichloë* and *Claviceps* were diverged around 58.8 MYA followed by horizontal transfer of the FTRL sequence into plant species after 39 MYA, the comparison of the *Claviceps* and plants combination include the diversification period in the *Epichloë* lineage (from 58.8 MYA to the HGT event), while the Triticeae and Poeae combination merely represent diversification between plant species.

	Diverged age (MYA)	Synonymous			Non-synonymous			Ka/Ks
		Ks (AVE)	SDV	Rate (Ks/million years)	Ka (average)	SDV	Rate (Ka/millon years)	
*Claviceps*-plants	58.8	1.0612	0.2560	0.0183	0.2307	0.0064	0.0040	0.2174
*Claviceps*-*Epichloë*	58.8	0.8517	0.1745	0.0147	0.1412	0.0073	0.0024	0.1657
Triticeae-Poeae	21	0.2767	0.0231	0.0132	0.1041	0.0008	0.0050	0.3760
*E. gansuensis*-other *Epichloë*	7.2	0.1435	0.0112	0.0199	0.0519	0.0055	0.0072	0.3619

**Figure 6:**
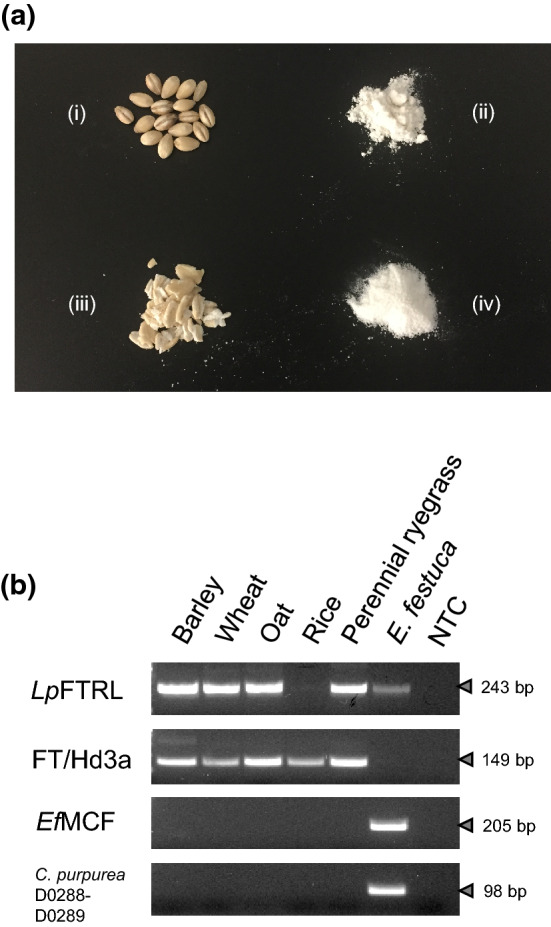
PCR-based detection of *Lp*FTRL-related gene from retail products. (**a**) Retail products of barley (i), wheat (ii), oat (iii), and rice (iv). (**b**) PCR assay results were visualised on an agarose gel, using the primers designed for *Lp*FTRL. The size (bp) of PCR amplicons is indicated on the right side of each image. As a control experiment, PCR primers for the florigen candidate gene (FT in wheat and barley, and Hd3a in rice) were used. For demonstration of absence of *Epichloë* and *Claviceps* species in the retail products, PCR primers specific to the fungal species (EfMCF_F and R, and C.purpurea_D0288F and D0289R) were used. The gDNA samples from the perennial ryegrass genotype Impact_04_ and *E. festucae* were used, as positive controls for amplification with the plant and fungus-specific primers, respectively. With the PCR primers specific to *Claviceps* species, amplification from *E. festucae* gDNA template was observed, presumably due to sequence similarity between *Epichloë* and *Claviceps* species. ‘NTC’ stands for ‘no DNA template control’.

Pearl barley, wheat plain flour, rolled oat, and rice flour products were obtained from retail shops, and DNA was extracted from the retail products. PCR primers for the *Lp*FTRL sequence within a conserved region between plant and *Epichloë* species were designed. Through PCR, the sequences corresponding to *Lp*FTRL were amplified from barley, wheat, oat, and *E. festuca* gnomic DNA (gDNA) templates, but not from rice, confirming the presence of the fungus-originated gene in Triticeae and Poeae cereal species (Fig. 6, Supplementary Information 12). A control experiment with fungi-specific primers confirmed absence of gDNA of *Epichloë* and *Claviceps* species, especially the rye ergot fungus, *C. purpurea*, in the retail products^24,29^.

## Discussion

In the current study, identification of fungus-originated genes from crop species has been reported. The possibility of DNA sequencing artifacts or contribution from microbiome has been excluded through a rigorous approach. The identification of plant repetitive element-like sequences in the perennial ryegrass genome contigs (GenBank UIs: MG680924 and MG680923) suggested that *Lp*FTRL and *Lp*DUF3632 are located in the plant genome. A BLASTN search for *Lp*FTRL indicated presence of putative orthologues in Tausch's goatgrass, barley, wheat, and cereal rye. Similarly, *Lp*DUF3632-like sequences were identified from SRA data of Italian ryegrass and tall fescue. However, no similarity hit was obtained from *Brachypodium*, rice, sorghum, *Zea Mays* (L.) and *A. thaliana*. The genome sequences and SRA data described above were generated at divergent institutes and organisations, and the lineage specificity of the genes, hence, suggested ancestral HGT, rather than that the two sequences were derived from DNA sequencing artifacts, mis-annotation or contributions of microorganisms.

Although the *in slico* screening suggested absence of an *FTRL*-related sequence in harding grass transcriptome, PCR amplification was observed from the harding grass gDNA template (Fig. 1). The tissue-specific expression pattern of *Lp*FTRL suggested a possibility that the corresponding sequence is present in the harding grass genome, and the expression level was, however, below the limit of detection in the leaf tissues, which were used for transcriptome sequencing. Therefore, it is likely that the *FTRL*-related sequence is also present in harding grass.

The phylogenetic analysis suggested a possibility that HGT events occurred after diversification of ancestral *Epichloë* species from the *Claviceps* lineage around 59 million years ago (MYA)^29^. Molecular taxonomy and the estimated divergence ages suggested that the two genes were transferred from the fungus lineage into plant species, but not vice versa. Based on the divergence age of the Brachypodieae tribe from other Pooideae species, the *FTRL* gene was predicted to have been transferred into plants after 32–39 MYA^30^. A formation of a cluster of the plant *FTRL* sequences suggested a single transfer event, rather than multiple events. It is, therefore, possible that the fungus gene was transferred into the chromosome 7 of a common ancestor of Triticeae and Poeae species, followed by gene duplication in barley genome. The *DUF3632*-like sequence is likely to have been transferred into a common ancestor of *Loliinae* species, independently of the FTR and β-glucanase(-like) gene, as the DNA-based marker for *Lp*BGNL has been assigned into perennial ryegrass LG 5^23^. The phylogenetic tree of the *DUF3632*(-like) genes suggested a possibility that the ancestral gene transfer event occurred after diversification of modern *Epichloë* species (7.2 MYA)^29^. These results indicated a possibility of at least three independent HGT events since 39 MYA. Compared with these three events, it is possible that the *Fhb7* gene in *T. elongatum* was transferred more recently. The *Fhb7* gene is likely to have been transferred after *T. elongatum* diverged from wheat, which was estimated to have been 4.77–4.96 MYA^26^. Comparison of genomic sequences including *DUF3632*-like genes suggested a transformation-like ancestral HGT event, due to conservation of the position of the putative intron between plant and *Epichloë* species. The transformation-like events have also been suggested for *Lp*BGNL and *Fhb7*^23,26^*.*

Identification of *Lp*FTRL facilitated a unique opportunity to compare nucleotide substitution rates between fungal and plant lineages, using the sequences diverged only after 59 MYA (the *Claviceps-Epichloë* divergence period). Interestingly, the Ks/million years ratios of the plant *FTRL* sequences and counterparts in fungi were not substantially different (Table 2). The Ka/Ks ratios for the plant *FTRL* (0.376) and corresponding *E. gansuensis*-other *Epichloë* sequences (0.3619) were relatively close, suggesting that both plant and fungi genes have been subjected to selection pressures. The Ka/million years and Ks/million years rates from the *E. gansuensis*-other *Epichloë* combination were higher than the other combinations, which could be partly attributed to the shortest divergence period (7.2 million years) in the tested combinations. The Ks and Ka values for *Lp*FTRL and *Lp*DUF3632 proposed that plant *FTRL* and *DUF3632* genes retain functionalities at the protein level, and the genes have contributed to natural adaptation of the plant species. The intron sequences of *Lp*DUF3632 were much less conserved than the exon sequences, likely due to lower levels of the pressure, which implies that the selective pressures have been an important factor for retaining of sequence similarity to the corresponding *Epichloë* sequences. This hypothesis suggests a possibility of occurrence of additional unrevealed ancient HGT events, of which transferred genes (or sequences) have, however, been eliminated from plant genomes during the subsequent evolutionary process, due to lack of adequate levels of the pressure. The close physical contacts between the host plants and symbiotic and/or pathogenic fungi could have facilitated the relatively frequent transfer events^21^. As both *Epichloë* and *Claviceps* species infect plant reproductive tissues^24,29^, the presence close to germ cells might have been an important factor of the multiple HGT events^31^.

The ‘natural’ transgene was detected from a range of grain products. Cultivation of barley, rye, and wheat, including emmer and einkorn wheat, began over 10,000 years ago, and domestication of oat was completed by around 3000 years ago^32,33^. Compared with those, GM technologies and products have substantially shorter histories, shorter than 50 years since the first report of genetic engineered bacteria, and 25 years since the first approval of GM commercial food products^34,35^. The public concerns have been one of major factors involved in the regulatory decisions^36,37^. The identification of the ‘natural’ transgenes in staple species with long cultivation histories, however, may increase public acceptance of GM and gene-edited crop products^19^. Lateral acquisitions of nuclear genes, especially from taxonomically distant species, have been rarely documented, and it has been believed that such ‘natural’ genetic modifications never happened in flowering plants^13^. With the previously reported ‘natural’ *Agrobacterium*-mediated conjugation(-like) events in tobacco and sweet potato^19^, the identification of transformation(-like) events questions the traditional scientific perception. Occurrence and frequency of HGT may have been largely depending on the closeness and frequencies of physical contacts of the gene-donor, typically microbiome and parasitic plants, to germ cells of the recipient plants^21,31^.

## Materials and methods

### Non-poly(A) tailed RNA sequencing

Fresh young leaves of an individual of perennial ryegrass cultivar Trojan were harvested. Total RNA including small molecules was extracted using the RNeasy Plant mini kit (QIAGEN) following the modified protocol of related products (Purification of miRNA from animal cells using the RNeasy Plus Mini Kit and RNeasy MinElute Cleanup Kit; https://www.qiagen.com/nl/resources/download.aspx?id=4a299ac7-4a55-4ae0-aaca-f3ae8f66d9c1&lang=en). A sequencing library was prepared using the Small RNA sequencing library preparation kit (New England Biolabs; NEB), and a fraction containing non-coding RNA molecules (140–150 bp in length, including sequencing adapters) was purified using the BluePippin platform (Sage Science). The products were characterised on the 2200 TapeStation instrument using the D1000 kit (Agilent). The library was loaded on the MiSeq platform (Illumina), following the manufacture’s instruction, and a sequencing analysis was performed with the MiSeq Reagent Kit v3 (150-cycle) kit (Illumina). The outcome data were processed with the FastX-tool-kit package (https://hannonlab.cshl.edu/fastx_toolkit/), to remove adapter sequence and generate the unique read dataset.

### BLAST-based screening

The transcriptome dataset of *E. festucae* (file name: M3 transcript sequences) was obtained from the website of Kentucky university (https://www.endophyte.uky.edu). The TSA (GFSR01000001-GFSR01044773) and non-poly(A) tailed RNA unique read datasets of perennial ryegrass were prepared for the DNA sequence homology search. The homology search was performed with the megablast function of the BLAST + package (https://blast.ncbi.nlm.nih.gov/Blast.cgi?PAGE_TYPE=BlastDocs&DOC_TYPE=Download), and the threshold E value was set at 1 e−10. The resulting data was imported into the Microsoft Excel software for the manual examination. The manual examination was performed using the NCBI BLAST tools (https://blast.ncbi.nlm.nih.gov/Blast.cgi).

The homologues sequence in representative flowering plant species were performed on the Ensembl Plants website (https://plants.ensembl.org/index.html). The *Lp*FTRL sequence was subjected to BLAST-based search against cereal rye SRA dataset (BioProject UI: PRJEB3229). The *Lp*DUF3632 sequence were subjected to the search against the following SRA datasets; Italian ryegrass (SRA UIs: SRX1604870 and SRX1604871), tall fescue (SRX1056957), orchard grass (ERX1842528 and SRX738187), Antarctic hairgrass (SRX465632), *Poa* species (SRX745831, SRX745855, and SRX745858), and harding grass (SRX669405).

### DNA sample preparation

Plant seeds were obtained from the Genetic Resources Unit of Institute for Biological Environmental and Rural Studies (IBERS; Aberystwyth, Wales, UK) and the South Australian Research and Development Institute (SARDI)^23^. The plants were germinated on a filter paper in a petri dish. gDNA was extracted from fresh leaf of each plant genotype using the DNeasy Plant mini kit (QIAGEN) following the manufacture’s instruction. gDNA was extracted from barley (pearled grains), wheat (plain flour), oat (rolled grains) and rice (flour) using the DNeasy Plant mini kit.

### PCR-based screening

PCR primers were designed using the Oligo Calc tools (https://biotools.nubic.northwestern.edu/OligoCalc.html). For cross-species amplification, primers were designed to generate short DNA fragments (< 251 bp in length) within highly conserved regions of the target sequences (Supplementary Information 13). The PCR was performed on the CFX Real-Time PCR Detection Systems (Bio-Rad), with the Luna Universal qPCR Master Mix (NEB), and data analysis was performed using the CFX Manager Software (Bio-Rad). Visualisation of PCR products was performed on the 2200 TapeStation instrument or on an agarose gel (2.0% w/v) stained with SYBR Green (Thermo Fisher) through electrophoresis.

### Validation of the PCR-based screening method

PCR primers were designed based on perennial ryegrass sequences to amplify larger fragments (> 900 bp), for use as DNA templates of a standard curve assay (SCA) (Supplementary Information 13). The PCR amplicons were amplified from the Impact_04_ genotype, using the MyTaq DNA Polymerase kit, and the amplicons were cleaned using the Monarch PCR & DNA Cleanup Kit (NEB). DNA concentration was adjusted to 1 pg/μl, and a tenfold series dilution (from 1 pg/μl to 1 × 10^−4^ pg/μl) was prepared. For fungus-specific primers, a fourfold series dilution (from 1 ng/μl to 1 × 4^−4^ ng/μl) of *E. festucae* gDNA was prepared. The SCA was performed on the CFX Real-Time PCR Detection Systems, with the Luna Universal qPCR Master Mix, followed by data analysis with the CFX Manager Software (Supplementary Information 14).

### Genetic linkage mapping

PCR primers (LpDUF3632_SCA_F and R in Supplementary Information 13) were used for amplification of an *Lp*DUF3632-related sequence from the C3 parental genotype of the p150/112 reference genetic mapping population^23^. A sequencing library for the MiSeq platform (Illumina) was prepared from the PCR amplicons, following the previously described MspJI-based method. The prepared library was characterised and quantified with the TapeStation and Qubit instruments (Thermo Fisher Scientific). The library was loaded on the MiSeq platform (Illumina), following the manufacture’s instruction, and the outcome reads were assembled with the Sequencher 5.0 program (Gene Codes), to find the single nucleotide substitutions between the C3 haplotypes. An additional reverse PCR primer (5′-AGCGTGCTTGCCAGCCTCTC-3′) was prepared and used with the LpDUF3632_SCA_F primer, for the PCR–RFLP assay. DNA fragments were amplified from each individual of the genetic mapping population and digested with the *Sac*II restriction enzyme (NEB). Genetic linkage analysis was performed through use of the JoinMAP application.

### In silico analysis

Gene structure prediction was performed using the FGENESH program of the Softberry website (https://www.softberry.com/berry.phtml) using the ‘Monocot plants’ parameter. Alignments of amino acid sequences were generated with the CLUSTALW program (https://www.genome.jp/tools/clustalw/) with the default parameters. Sequence homology search was performed with the NCBI (https://www.ncbi.nlm.nih.gov/) and PredictProtein (https://open.predictprotein.org/) websites. Amino acid and DNA sequences were prepared for phylogenetic analysis with the MEGA7 program (https://www.megasoftware.net/) (Supplementary Information 15 and 16). Figure legends generated with the program can be found in Supplementary Information 17. Non-synonymous and synonymous nucleotide substitution ratio was calculated using the MEGA7 program. For gene expression analysis, in-house transcriptome read datasets were prepared through filtering of the transcriptome sequencing reads from Impact_04_ tissues using Impact_04_ genome contigs (> 999 bp). The number of reads which contained *Lp*FTRL or *Lp*DUF3632 sequences (no sequence mismatch for 60 bp or longer) were counted. For the gene expression analysis with the SRA data from Massey University (NCBI BioProject UI: PRJNA292034), the NCBI BLASTn function was used, with the megablast algorithm and word size of 64.

## Supplementary information

Supplementary Legends.

Supplementary Information 1.

Supplementary Information 2.

Supplementary Information 3.

Supplementary Information 4.

Supplementary Information 5.

## Data Availability

A part of data generated or analysed during this study are included in the published article and supplementary information files. The rest of datasets is available from the corresponding author on reasonable request.
