# Supplementary material for: Fungus-originated genes in the genomes of cereal and pasture grasses acquired through ancient lateral transfer
Source: Sci Rep. 2020 Nov 16;10:19883. doi: 10.1038/s41598-020-76478-4 (PMC7670438; doi:10.1038/s41598-020-76478-4)
Supplement: Supplementary file 1 — Supplementary Legends. [file 41598_2020_76478_MOESM6_ESM.docx]

# Supplementary Information legends

**Supplementary Information 1**

Unique sequencing reads from perennial ryegrass non-poly(A) tailed RNA

**Supplementary Information 2**

Sequence similarity search result between the perennial ryegrass TSA and *E.* *festucae* transcriptome data. Following to the BLAST-based analysis, the manual examination was performed using the NCBI BLAST function, through which the function of each *E.* *festucae* gene was predicted, and the similarity hits were categorised into ‘low homology’, ‘highly conserved’, ‘contamination’, or ‘confident candidate’. The similarity hits of which bit scores were lower than 200 were concluded as low homology hits (‘low homology’). Those showed >99% identity to the corresponding *E.* *festucae* gene, or a higher identity to other fungal species than *Epichloë* species were, then, categorised as contamination from plant-related microbiome (‘contamination’). The similarity hits related to genes which are highly conserved between eukaryote species were concluded as highly conserved gene-related hits (‘highly conserved’). The similarity hits which were not categorised into ‘low homology’, ‘highly conserved’, or ‘contamination’ were concluded as high confident HGT candidates (‘confident candidate’).

**Supplementary Information 3**

Sequence similarity search result between the perennial ryegrass non-poly(A) tailed RNA sequence and *E.* *festucae* transcriptome data. Following to the BLAST-based analysis, the manual examination was performed using the NCBI BLAST function, through which the function of each *E.* *festucae* gene was predicted, and the similarity hits were categorised into ‘low homology’, ‘highly conserved’, ‘contamination’, or ‘confident candidate’. The similarity hits, of which bit scores were lower than 70, were concluded as low homology hits (‘low homology’). Those showed >99% identity to the corresponding *E.* *festucae* gene, or a higher identity to other fungal species than *Epichloë* species were, then, categorised as contamination from plant-related microbiome (‘contamination’). The similarity hits related to genes which are highly conserved between eukaryote species were concluded as highly conserved gene-related hits (‘highly conserved’). The similarity hits which were not categorised into ‘low homology’, ‘highly conserved’, or ‘contamination’ were concluded as high confident HGT candidates (‘confident candidate’).

**Supplementary Information 4**

DNA and amino acid sequence alignments between HGT candidates and corresponding *E. festucae* sequences. The DNA sequence alignment for *Lp*FTRL(a) and *Lp*DUF3632(b), and amino acid sequent alignment for the predicted *Lp*FTRL(c) and *Lp*DUF3632(d) gene products were generated with the CLUSTALW program (https://www.genome.jp/tools-bin/clustalw). Conserved nucleotides are show with an asterisk (*), and dash (-) in the DNA sequences shows a gap (a and b). Conserved and semi- conserved amino acids are shown with an asterisk (*), colon (:), or period (.), depending on ‘conserved amino acid residues’, ‘including conserved substitution’ and ‘including semi-conserved substitution’, respectively, and a dash (-) in the amino acid sequences shows a gap (c and d). As the genomic region encoding EfM3.066060 gene from the *E. festucae* strain E2368 has not been completely assembled, the corresponding gene from the *E. festucae* strain Fl1 was used for alignment with *Lp*FTRL.

**Supplementary Information 5**

Result of the DNA sequence similarity search for perennial ryegrass HGT candidates. The SRA datasets were subjected to the similarity search using the ‘more dissimilar sequences (discontiguous megablast)’ parameter. Institute/Organisation denotes the research body from which the SRA dataset(s) were submitted. ‘N.S.’ stands for ‘no significant hits’. Although a single sequence similarity hit between *Lp*DUF3632 and supina bluegrass transcriptome data, which is indicated with a single asterisk(*), was obtained, supina bluegrass was not concluded to have an *Lp*DUF3632 orthologue, due to the low sequence similarity. For *Lp*FTRL, only a low sequence similarity hit and no hit were obtained from early meadow-grass and harding grass transcriptome data, respectively, which are indicated with double asterisk(**). The subsequent PCR-based screening, however, suggested the presence of *Lp*FTRL orthologues in harding grass (further details can be found in Supplementary Information 6). A control analysis was performed, using perennial ryegrass *Lp*BGNL and *E. festucae* *Ef*MCF sequences.

**Supplementary Information 6**

Cross-species PCR amplification result using *Lp*FTRL and *Lp*DUF3632 primers. (a) PCR amplification plot using perennial ryegrass, darnel, tall fescue, sheep fescue, orchard grass, and harding grass gDNA as templates. (b) Electrophoresis result of the PCR products. The upper and lower size-standard markers of the TapeStation D1000 kit were indicated with blue and green arrows, respectively. The target fragments were shown with a red arrow. The length of size ladder is shown on the left side of the electrophoresis result using the *Lp*DUF3632 primers. NTC stands for no-template control, in which molecular biology grade water was used, instead of a gDNA sample.

**Supplementary Information 7**

Genotyping of the p150/112 genetic mapping population for genetic linkage mapping of *Lp*DUF3632. (a) DNA sequence alignment of two *Lp*DUF3632-related haplotypes (C3_hap1 and 2) from the C3 parental genotype of the p150/112 genetic mapping population. An asterisk (*) denotes a conserved nucleotide between the two haplotypes, and the recognition site (CCGCGG) of the *Sac*II restriction enzyme in the C3_hap1 sequence was indicated with an underline. The sequences corresponding to PCR primers used for the PCR-RFLP assay are indicated with arrows. The expected size of PCR amplicons is 272 bp, and those of *Sac*II-digested products of the C3_hap1 sequence are 53 and 217 bp with a 2-bp cohesive end. (b) Visualised *Sac*II-treated PCR amplicons on a 2% agarose gel. The EasyLadder I (Bioline) was used as a size standard, and the 500, 250, and 100-bp fragments are indicated with red arrows. The number indicates genotype number of the genetic mapping population, and NTC stands for ‘no-template control’ for PCR, in which molecular biology grade water was used, instead of a DNA sample. The samples, with which the PCR-RFLP assay was not successful, are shown with an asterisk (*).

**Supplementary Information 8**

Read count-based gene expression analysis for *Lp*FTRL and *Lp*DUF3632.

**Supplementary Information 9**

Read count-based gene expression analysis for *Lp*FTRL and *Lp*DUF3632, and corresponding *E. festucae* EfM3.066060 and EfM3.028800 genes, using endophyte-devoted (E^-^) and -infected (E^+^) perennial ryegrass individuals.

**Supplementary Information 10**

Time course read count-based gene expression analysis for *Lp*FTRL and *Lp*DUF3632, and corresponding *E. festucae* EfM3.066060 and EfM3.028800 genes, using perennial ryegrass seeds and young seedlings. Time shows the period since the germination treatment. ‘N.A.’ denotes ‘not analysed’.

**Supplementary Information 11**

Nucleotide substitution ratio between *Lp*DUF3632 and EfM3.028800 (*E. festucae*). (a) DNA sequence alignment of the two genes. (b) Ks, Ka, and Ka/Ks ratio between the two genes.

**Supplementary Information 12**

Figure preparation process for the PCR-based screening of *Lp*FTRL-related gene. In the original image, the 100 bp DNA Ladder (NEB) was used as size standards (left lane), and samples unrelated to the current study were included (right lane). The image parts used for Figure 6b were indicated with red dashed squares, and corresponding parts in Figure 6b are indicated with red arrows.

**Supplementary Information 13**

PCR primers used in the current study.

**Supplementary Information 14**

Standard curve assay for validation of PCR-based screening. Amplification efficiencies (Es) were obtained using the PCR primers designed for *Lp*FTRL(a), *Lp*DUF3632(b), and *Ef*mcf(c). An assay was performed using the PCR primers designed for Triticeae/Poeae and *Epichloë* *FTRL* genes, using *E. festucae* gDNA as template(d). For each dilution point, 3 technical replications were performed, and a subsequent melt curve assay was performed. From the SCA for the plant/fungus-conserved *FTRL* sequence(d), the plots from the three 1 x 4^-4^ ng/μl samples and one 1 x 4^-3^ ng/μl sample were excluded, due to amplification of non-specific DNA fragments.

**Supplementary Information 15**

Amino acid sequences predicted from *Lp*FTRL and related genes.

**Supplementary Information 16**

Amino acid sequences predicted from *Lp*DUF3632 and related genes.

**Supplementary Information 17**

Legends for the phylogenetic trees in Figure 2. The legends for the phylogenetic trees including *Lp*FTRL(i) and *Lp*DUF3632(ii) were generated with the MEGA7 program.
