# Supplementary material for: Fungus-originated genes in the genomes of cereal and pasture grasses acquired through ancient lateral transfer
Source: Sci Rep. 2020 Nov 16;10:19883. doi: 10.1038/s41598-020-76478-4 (PMC7670438; doi:10.1038/s41598-020-76478-4)
Supplement: Supplementary file 2 — Supplementary Information 1. [file 41598_2020_76478_MOESM1_ESM.pdf]

Fungus-originated genes in the genomes of cereal and pasture grasses acquired through ancient lateral transfer

Hiroshi Shinozuka[1 *], Maiko Shinozuka[1], Ellen M. de Vries[1,2], Timothy I. Sawbridge[1,2], German C. Spangenberg[1,2], Benjamin G. Cocks[1,2]

[1]Agriculture Victoria, AgriBio, Centre for AgriBioscience, Bundoora, Victoria 3086, Australia
[2]School of Applied Systems Biology, La Trobe University, Bundoora, Victoria 3086, Australia
*Corresponding author: e-mail – hiroshi.shinozuka@agriculture.vic.gov.au

Supplementary Information 1

(supplied as a separate zipped FASTA file)

Supplementary Information 2

UI	Query sequence (*E. festuca* mRNA UI)	Predicted gene function	Subject sequence (Imp04 sRNA UI)	Percentage of identical matches	Alignment length	Number of mismatches	Number of gap openings	Start of alignment in query	End of alignment in query	Start of alignment in subject	End of alignment in subject	Expect value	Bit score	Conclusion
1	EfM3.002130.mRNA-1	4-monooxygenase	ID_150587_scaf76989_Loc_69787_0_59.6	94.872	39	2	0	1394	1432	452	414	2.68E-08	62.1	Low homology
2	EfM3.004490.mRNA-1	Hypothetical protein	ID_163699_scaf9194_Loc_2608_1_92.6	97.143	35	1	0	816	850	3184	3218	5.35E-08	60.2	Low homology
			ID_163699_scaf9194_Loc_2608_1_92.6	94.737	38	2	0	812	849	3192	3229	5.35E-08	60.2	Low homology
			ID_163699_scaf9194_Loc_2608_1_92.6	94.444	36	2	0	815	850	3189	3224	6.92E-07	56.5	Low homology
			ID_167702_Loc_45308_Contig1	100	30	0	0	821	850	2525	2554	6.92E-07	56.5	Low homology
			ID_103095_scaf24154_Loc_9220_0_2.9	100	30	0	0	821	850	151	122	6.92E-07	56.5	Low homology
			ID_167277_Loc_5323_Contig1	100	30	0	0	821	850	4216	4187	6.92E-07	56.5	Low homology
			ID_52024_C1113627_5.0	100	29	0	0	822	850	233	261	2.49E-06	54.7	Low homology
			ID_159054_Loc_42462_Contig1	96.875	32	1	0	818	849	415	446	2.49E-06	54.7	Low homology
3	EfM3.005710.mRNA-1	Ribosomal protein	ID_73315_scaf75000_Loc_65890_0_50.0	88.333	120	14	0	1	120	257	376	5.01E-34	145	Low homology
			ID_72969_C1205118_56.0	85.833	120	17	0	1	120	260	379	5.05E-29	128	Low homology
			ID_21513_C471496_63.0	83.333	120	19	1	109	227	1	120	1.83E-23	110	Low homology
4	EfM3.006320.mRNA-1	Zinc finger domain-containing protein	ID_139396_scaf3378_Loc_925_0_55.7	100	30	0	0	424	453	94	123	1.17E-06	56.5	Low homology
5	EfM3.006830.mRNA-1	evh1 (WASP-like)	ID_147337_scaf15771_Loc_4849_0_54.3	96.875	32	1	0	787	818	1493	1462	2.72E-06	54.7	Low homology
			ID_167844_Loc_30599_Contig2	100	28	0	0	791	818	3100	3073	9.79E-06	52.8	Low homology
			ID_158547_scaf59212_Loc_41218_2_21.0	100	28	0	0	794	821	2211	2184	9.79E-06	52.8	Low homology
6	EfM3.008010.mRNA-1	Tubulin alpha-1 chain	ID_107380_C1339233_76.0	75	300	70	5	988	1284	599	302	3.97E-30	134	Low homology
7	EfM3.009620.mRNA-1	Co-chaperone	ID_149203_scaf18006_Loc_5763_0_69.6	100	30	0	0	1421	1450	228	199	9.65E-07	56.5	Low homology
8	EfM3.011820.mRNA-1	LteA	ID_159000_Loc_7411_Contig1	86.364	66	9	0	1272	1337	1270	1205	1.51E-11	73.1	Low homology
9	EfM3.014010.mRNA-1	Tubulin beta-1 chain	ID_151864_scaf44786_Loc_25252_4_58.4	77.504	1178	247	16	115	1283	1597	429	0	691	Highly conserved
10	EfM3.015230.mRNA-1	Serine/threonine-protein phosphatase PP1	ID_151594_scaf17824_Loc_5685_2_70.9	75.221	678	158	10	409	1081	1534	862	4.91E-84	313	Highly conserved
			ID_146452_C1443794_47.0	72.2	741	181	19	467	1193	444	1173	3.09E-51	204	Highly conserved
			ID_151594_scaf17824_Loc_5685_2_70.9	75.221	678	158	10	76	748	1534	862	3.43E-84	313	Highly conserved
			ID_146452_C1443794_47.0	72.258	775	188	21	134	893	444	1206	9.96E-55	215	Highly conserved
11	EfM3.017210.mRNA-1	(Unknown)	ID_151492_scaf90288_Loc_102085_1_48.9	100	30	0	0	295	324	112	83	9.38E-07	56.5	Low homology
12	EfM3.017740.mRNA-1	Atin	ID_137039_scaf104713_Loc_161785_0_24.0	81.485	1118	200	7	15	1128	1143	29	0	913	Highly conserved
			ID_146511_C1443854_51.0	80.41	1123	206	12	13	1128	1542	427	0	843	Highly conserved
			ID_152894_Loc_4366_Contig1	76.396	1110	234	25	31	1126	399	1494	1.64E-162	573	Highly conserved
			ID_144000_scaf99282_Loc_134638_0_16.5	84.768	453	67	2	4	455	51	502	2.23E-126	453	Highly conserved
			ID_138819_Loc_22558_Contig1	78.069	611	132	2	31	640	283	892	8.25E-106	385	Highly conserved
			ID_38036_C1047005_3.0	82.988	241	39	2	43	282	240	1	3.19E-55	217	Highly conserved
13	EfM3.018520.mRNA-1	Heat shock protein	ID_156631_C1454200_13.0	76.503	183	43	0	487	669	894	1076	6.18E-20	100	Low homology
14	EfM3.020300.mRNA-1	60S ribosomal protein	ID_130248_scaf65103_Loc_49504_0_55.4	78.729	362	63	12	408	762	700	346	4.07E-59	230	Highly conserved
			ID_122559_scaf44180_Loc_24685_0_50.8	74.652	359	81	10	408	761	585	232	3.26E-35	150	Low homology
15	EfM3.020690.mRNA-1	30S ribosomal protein	ID_100824_C1315633_5.0	100	544	0	0	449	992	1	544	0	1005	Contamination
16	EfM3.020760.mRNA-1	TATA-box binding protein	ID_155007_C1452728_14.0	100	762	0	0	1	762	1421	660	0	1408	Contamination
17	EfM3.021070.mRNA-1	(Low homology)	ID_167085_scaf35606_Loc_17362_2_72.5	100	28	0	0	549	576	2821	2794	7.13E-06	52.8	Low homology
18	EfM3.021690.mRNA-1	Elongation factor 1-alpha	ID_37130_C1042903_61.0	80.443	271	47	6	211	478	3	270	1.10E-50	202	Highly conserved
19	EfM3.022140.mRNA-1	Histone H3	ID_111236_C1352867_53.0	100	648	0	0	23	670	1	648	0	1197	Contamination
20	EfM3.023770.mRNA-1	Taurine catabolism dioxygenase	ID_162284_scaf105655_Loc_167547_0_41.8	100	29	0	0	1077	1105	2820	2848	3.64E-06	54.7	Low homology
21	EfM3.026100.mRNA-1	Histone 2B	ID_118297_C1376310_36.0	100	745	0	0	54	798	1	745	0	1376	Contamination
22	EfM3.027570.mRNA-1	(Unknown)	ID_130735_Loc_954_Contig1	100	30	0	0	172	201	204	233	1.34E-06	56.5	Low homology

Supplementary Information 2 (Cont'd)

UI	Query sequence (*E. festuca* mRNA UI)	Predicted gene function	Subject sequence (Imp04 sRNA UI)	Percentage of identical matches	Alignment length	Number of mismatches	Number of gap openings	Start of alignment in query	End of alignment in query	Start of alignment in subject	End of alignment in subject	Expect value	Bit score	Conclusion
23	EfM3.029970.mRNA-1	Glutamate decarboxylase	ID_147008_scaf32841_Loc_15378_3_61.9	73.934	422	83	21	359	761	1254	841	1.73E-33	145	Low homology
24	EfM3.030740.mRNA-1	14-3-3 protein	ID_152642_Loc_26578_Contig1	73.56	677	147	25	45	702	212	875	2.99E-59	230	Highly conserved
			ID_137296_C1427890_79.0	72.652	607	149	13	112	711	1000	404	6.56E-46	185	Low homology
25	EfM3.031060.mRNA-1	Epichloe glyceriae NsfA (nsfA) gene	ID_122431_C1388982_43.0	100	30	0	0	222	251	225	196	3.92E-07	56.5	Low homology
26	EfM3.031160.mRNA-1	ADP/ATP carrier protein	ID_154190_C1452060_66.0	76.357	884	189	16	51	924	537	1410	1.44E-127	457	Highly conserved
27	EfM3.031590.mRNA-1	60S ribosomal protein	ID_61837_C1158073_64.0	83.333	174	27	2	225	397	79	251	3.20E-38	159	Low homology
28	EfM3.031670.mRNA-1	Carotenoid cleavage dioxygenase-like	ID_164022_scaf110987_Loc_212424_0_62.0	100	29	0	0	710	738	1847	1819	4.88E-06	54.7	Low homology
29	EfM3.033810.mRNA-1	(Unknown)	ID_164911_Loc_25060_Contig1	97.297	37	1	0	2833	2869	3498	3462	1.33E-08	63.9	Low homology
30	EfM3.034100.mRNA-1	Cyclophilin-like peptidylprolyl cis- trans isomerase	ID_154764_scaf49891_Loc_30334_4_31.1	89.091	55	6	0	364	418	519	573	8.86E-11	69.4	Low homology
31	EfM3.034390.mRNA-1	40S ribosomal protein	ID_61706_C1157385_4.0	86.095	338	43	4	348	683	1	336	9.59E-99	361	Highly conserved
31*	EfM3.034390.mRNA-2	40S ribosomal protein	ID_61706_C1157385_4.0	86.095	338	43	4	111	446	1	336	6.62E-99	361	Highly conserved
32	EfM3.037260.mRNA-1	Fumarylacetoacetase	ID_158676_scaf72866_Loc_62190_4_56.6	90	60	4	2	1560	1618	1654	1596	1.50E-12	76.8	Low homology
33	EfM3.038220.mRNA-1	Unknown protein gene	ID_61576_C1156845_7.0	100	331	0	0	1	331	8	338	2.58E-174	612	Contamination
34	EfM3.041850.mRNA-1	(Unknown)	ID_143146_scaf26364_Loc_10607_0_57.5	96.97	33	1	0	619	651	499	467	1.86E-06	56.5	Low homology
35	EfM3.043150.mRNA-1	Short-chain dehydrogenase/reductase family protein	ID_100956_C1316099_5.0	96.97	33	1	0	396	428	113	81	6.88E-07	56.5	Low homology
36	EfM3.043630.mRNA-1	Ubiquitin	ID_132283_scaf104602_Loc_161137_1_55.3	80.406	689	133	2	229	916	250	937	1.36E-147	523	Highly conserved
			ID_132283_scaf104602_Loc_161137_1_55.3	85.055	455	68	0	1	455	478	932	8.34E-130	464	Highly conserved
			ID_96316_C1298362_5.0	83.521	443	73	0	1	443	65	507	8.52E-115	414	Highly conserved
			ID_96316_C1298362_5.0	85.116	215	32	0	1	215	293	507	1.99E-56	220	Highly conserved
			ID_24665_C973557_85.0	84.711	242	37	0	253	494	244	3	4.26E-63	243	Highly conserved
			ID_24665_C973557_85.0	97.368	38	1	0	1	38	40	3	9.89E-10	65.8	Low homology
			ID_24165_C984173_63.0	79.352	247	47	4	430	674	245	1	2.04E-41	171	Low homology
			ID_22191_C576878_63.0	86.452	155	21	0	1	155	157	3	2.04E-41	171	Low homology
			ID_73315_scaf75000_Loc_65890_0_50.0	86.429	140	19	0	209	348	237	376	2.05E-36	154	Low homology
			ID_23926_C932263_63.0	87.5	120	15	0	1	120	120	1	5.74E-32	139	Low homology
			ID_72969_C1205118_56.0	86.667	120	16	0	229	348	260	379	2.67E-30	134	Low homology
			ID_24711_C562020_90.0	79.63	162	31	2	421	581	2	162	9.68E-25	115	Low homology
37	EfM3.043740.mRNA-1	Glutamate synthase precursor	ID_166964_C1460095_67.0	92.5	40	3	0	3261	3300	2428	2389	1.22E-06	58.4	Low homology
38	EfM3.044970.mRNA-1	Phosphatidylinositol transporter	ID_164192_scaf63216_Loc_46889_0_70.3	100	29	0	0	214	242	3665	3637	4.06E-06	54.7	Low homology
			ID_129941_scaf12342_Loc_3586_2_38.0	100	29	0	0	214	242	587	559	4.06E-06	54.7	Low homology
39	EfM3.049140.mRNA-1	Ketol-acid reductoisomerase, mitochondrial precursor	ID_142961_C1438482_12.0	99.794	1458	3	0	543	2000	1	1458	0	2676	Highly conserved
40	EfM3.056300.mRNA-1	Ubiquitin	ID_129560_scaf88696_Loc_97480_1_23.9_FORK	100	512	0	0	85	596	584	73	0	946	Contamination
			ID_129560_scaf88696_Loc_97480_1_23.9_FORK	100	44	0	0	1	44	725	682	6.29E-15	82.4	Low homology
			ID_73913_C1208804_79.0	83.817	241	39	0	178	418	383	143	2.12E-59	230	Highly conserved
			ID_24165_C984173_63.0	85.388	219	30	2	92	309	218	1	2.75E-58	226	Highly conserved
			ID_23734_C883576_63.0	87.97	133	16	0	92	224	133	1	1.02E-37	158	Low homology
			ID_28873_C1004543_63.0	88.288	111	13	0	92	202	146	256	1.71E-30	134	Low homology
			ID_24711_C562020_90.0	85.484	124	14	4	92	213	38	159	2.86E-28	126	Low homology
41	EfM3.066060.partial-1.mRNA-1	(Unknown)	ID_131748_scaf73966_Loc_64153_0_8.4	100	463	0	0	594	1056	192	654	0	856	Contamination
			ID_131748_scaf73966_Loc_64153_0_8.4	99.476	191	0	1	458	648	1	190	3.64E-94	346	Contamination
42	EfM3.072910.mRNA-1	18S ribosomal RNA gene	ID_167780_scaf52933_Loc_33715_0_62.2	73.364	214	44	11	1	205	4559	4350	9.57E-11	67.6	Low homology
43	EfM3.073330.mRNA-1	Hypothetical protein	ID_167085_scaf35606_Loc_17362_2_72.5	100	29	0	0	5	33	2793	2821	2.00E-06	54.7	Low homology
44	EfM3.073660.mRNA-1	(Unknown)	ID_153921_scaf80986_Loc_78244_1_63.6	100	29	0	0	213	241	650	678	6.13E-07	54.7	Low homology
45	EfM3.066060.partial-2.mRNA-1*		ID_150936_C1449060_17.0*	83.996	906	121	11	1	903	759	1643	0	848	Confident candidate (FTRL)
46	EfM3.079120.mRNA-1	Nuclear protein Qri2/Nse4	ID_145242_Loc_145021_Contig1	94.444	36	2	0	200	235	53	18	1.17E-06	56.5	Low homology
47	EfM3.082260.mRNA-1	Heat shock protein	ID_156210_C1453822_27.0	100	1971	0	0	1	1971	143	2113	0	3640	Contamination

Supplementary Information 3

UI	Query sequence (E. festuca mRNA UI)	Predicted gene function	Subject sequence (Imp04 sRNA UI)	Percentage of identical matches	Alignment length	Number of mismatches	Number of gap openings	Start of alignment in query	End of alignment in query	Start of alignment in subject	End of alignment in subject	Expect value	Bit score	Conclusion
1	EfM3.013890.mRNA-1	Bete glucanase	1370893-1	95.868	121	5	0	506	626	1	121	9.28E-49	196	Confident candidate (BGNL)
			309980-1	95.798	119	5	0	508	626	1	119	1.20E-47	193	Confident candidate (BGNL)
			702507-1	96.667	60	2	0	123	182	55	114	7.48E-20	100	Confident candidate (BGNL)
2	EfM3.017740.mRNA-1	Actin	1060002-1	85.606	132	19	0	208	339	1	132	1.13E-31	139	Highly conserved
3	EfM3.021690.mRNA-1	Elongation factor 1-alpha	895107-1	80.357	112	20	2	314	424	1	111	6.55E-15	84.2	Highly conserved
			1289249-1	92.683	41	3	0	361	401	18	58	1.10E-07	60.2	Low homology
			298915-1	92.683	41	3	0	361	401	18	58	1.10E-07	60.2	Low homology
4	EfM3.028800.mRNA-1	DUF3632	734684-1	95.588	68	3	0	717	784	17	84	8.53E-23	110	Confident candidate (DUF3632)
5	EfM3.029790.mRNA-1	Epichloenin A synthetase (sidN) gene	259443-1	100	96	0	0	5080	5175	96	1	2.95E-42	178	Contamination
6	EfM3.030740.mRNA-1	14-3-3 protein	214533-2	97.872	47	1	0	170	216	12	58	1.42E-14	82.4	Highly conserved
			740745-1	85.526	76	11	0	141	216	3	78	5.10E-14	80.5	Highly conserved
			1135617-1	78.431	102	22	0	172	273	21	122	3.97E-10	67.6	Highly conserved
			277525-1	94.872	39	2	0	178	216	1	39	1.85E-08	62.1	Highly conserved
7	EfM3.031160.mRNA-1	ADP/ATP carrier protein	713946-1	86.607	112	15	0	751	862	1	112	2.66E-27	124	Highly conserved
			548758-1	82.609	115	19	1	668	782	4	117	4.48E-20	100	Highly conserved
8	EfM3.031670.mRNA-1	Carotenoid cleavage dioxygenase-like	1139267-1	97.143	35	1	0	704	738	10	44	1.66E-07	60.2	Low homology
			1139267-1	96.97	33	1	0	710	742	10	42	2.15E-06	56.5	Low homology
			1112764-1	97.143	35	1	0	704	738	10	44	1.66E-07	60.2	Low homology
			1112764-1	96.97	33	1	0	710	742	10	42	2.15E-06	56.5	Low homology
9	EfM3.033720.mRNA-1	Small nucleolar ribonucleoprotein complex subunit	893956-1	89.916	119	12	0	81	199	119	1	1.16E-35	154	Highly conserved
10	EfM3.034100.mRNA-1	Cyclophilin-like peptidylprolyl cis- trans isomerase	1188904-1	89.091	55	6	0	364	418	37	91	1.42E-10	69.4	Low homology
			163280-2	89.091	55	6	0	364	418	27	81	1.42E-10	69.4	Low homology
			770863-1	100	30	0	0	389	418	27	56	1.10E-06	56.5	Low homology
11	EfM3.043630.mRNA-1	Ubiquitin	1361705-1	88	125	14	1	108	232	1	124	5.49E-34	147	Highly conserved
			861604-1	90.526	95	9	0	400	494	3	97	7.15E-28	126	Highly conserved
			723199-1	84.034	119	19	0	400	518	7	125	1.55E-24	115	Highly conserved
			723199-1	87.692	65	8	0	1	65	64	128	7.31E-13	76.8	Highly conserved
			1099541-1	87	100	13	0	1	100	19	118	5.57E-24	113	Highly conserved
			445270-1	85.577	104	15	0	400	503	4	107	7.20E-23	110	Highly conserved
			445270-1	97.368	38	1	0	1	38	61	98	1.58E-09	65.8	Low homology
			207354-2	87.368	95	12	0	1	95	36	130	7.20E-23	110	Highly conserved
			207354-2	79.508	122	25	0	424	545	3	124	3.38E-16	87.9	Highly conserved
			1329787-1	86.957	92	12	0	400	491	2	93	3.35E-21	104	Highly conserved
			583427-1	86.957	92	12	0	400	491	4	95	3.35E-21	104	Highly conserved
			319749-1	85.714	98	14	0	394	491	1	98	3.35E-21	104	Highly conserved
			704887-1	90.667	75	7	0	1	75	32	106	4.34E-20	100	Highly conserved
			1101849-1	87.654	81	10	0	1	81	63	143	2.02E-18	95.3	Highly conserved
			507841-1	92.188	64	5	0	1	64	61	124	2.61E-17	91.6	Highly conserved
			810975-1	90.769	65	6	0	1	65	54	118	3.38E-16	87.9	Highly conserved
			966624-1	87.671	73	9	0	400	472	3	75	1.21E-15	86.1	Highly conserved
			559944-1	93.103	58	4	0	256	313	3	60	1.21E-15	86.1	Highly conserved
			1297161-1	94	50	3	0	1	50	54	103	7.31E-13	76.8	Highly conserved
			169205-2	80.208	96	19	0	442	537	3	98	9.45E-12	73.1	Highly conserved
			1345902-1	97.368	38	1	0	1	38	32	69	1.58E-09	65.8	Low homology
			1112182-1	93.182	44	3	0	448	491	35	78	1.58E-09	65.8	Low homology
12	EfM3.043740.mRNA-1	Glutamate synthase precursor	1120757-1	92.5	40	3	0	3261	3300	78	39	1.89E-06	58.4	Low homology
			1015117-1	92.5	40	3	0	3261	3300	13	52	1.89E-06	58.4	Low homology
			795997-1	92.5	40	3	0	3261	3300	77	116	1.89E-06	58.4	Low homology
			716534-1	92.5	40	3	0	3261	3300	65	104	1.89E-06	58.4	Low homology
			715506-1	92.5	40	3	0	3261	3300	36	75	1.89E-06	58.4	Low homology
13	EfM3.046540.mRNA-1	AP-1 complex subunit	1115250-1	91.525	59	5	0	728	786	1	59	3.17E-14	82.4	Highly conserved
14	EfM3.055400.mRNA-1	Hypothetical protein	893956-1	82.114	123	14	7	205	323	1	119	1.68E-19	99	Contamination
15	EfM3.056300.mRNA-1	Ubiquitin	1235442-1	91.667	108	7	2	175	281	1	107	9.75E-35	148	Highly conserved
			1139238-1	87.85	107	13	0	152	258	3	109	4.57E-28	126	Highly conserved
16	EfM3.059200.mRNA-1	Dihydropteroate synthase	893956-1	80.645	124	14	8	99	217	1	119	6.60E-16	87.9	Contamination

Supplementary Information 3 (Cont'd)

UI	Query sequence (E. festuca mRNA UI)	Predicted gene function	Subject sequence (Imp04 sRNA UI)	Percentage of identical matches	Alignment length	Number of mismatches	Number of gap openings	Start of alignment in query	End of alignment in query	Start of alignment in subject	End of alignment in subject	Expect value	Bit score	Conclusion
17	EfM3.072910.mRNA-1	18S ribosomal RNA gene	1412826-1	100	31	0	0	175	205	26	56	9.29E-08	58.4	Low homology
			1384143-1	100	31	0	0	175	205	33	3	9.29E-08	58.4	Low homology
			1384134-1	100	31	0	0	175	205	33	3	9.29E-08	58.4	Low homology
			1382736-1	100	31	0	0	175	205	70	40	9.29E-08	58.4	Low homology
			1314446-1	100	31	0	0	175	205	39	9	9.29E-08	58.4	Low homology
			1308423-1	100	31	0	0	175	205	69	39	9.29E-08	58.4	Low homology
			1293297-1	100	31	0	0	175	205	51	21	9.29E-08	58.4	Low homology
			1258061-1	100	31	0	0	175	205	49	19	9.29E-08	58.4	Low homology
			1257979-1	100	31	0	0	175	205	65	35	9.29E-08	58.4	Low homology
			1231567-1	100	31	0	0	175	205	53	23	9.29E-08	58.4	Low homology
			1200621-1	100	31	0	0	175	205	91	61	9.29E-08	58.4	Low homology
			1180933-1	100	31	0	0	175	205	69	39	9.29E-08	58.4	Low homology
			1140399-1	100	31	0	0	175	205	96	66	9.29E-08	58.4	Low homology
			1127019-1	100	31	0	0	175	205	48	18	9.29E-08	58.4	Low homology
			1051157-1	78.824	85	18	0	1	85	44	128	9.29E-08	58.4	Low homology
			894966-1	100	31	0	0	175	205	31	1	9.29E-08	58.4	Low homology
			878595-1	100	31	0	0	175	205	41	11	9.29E-08	58.4	Low homology
			857564-1	100	31	0	0	175	205	78	48	9.29E-08	58.4	Low homology
			845146-1	100	31	0	0	175	205	53	23	9.29E-08	58.4	Low homology
			831825-1	100	31	0	0	175	205	85	55	9.29E-08	58.4	Low homology
			714515-1	100	31	0	0	175	205	37	7	9.29E-08	58.4	Low homology
			632619-1	100	31	0	0	175	205	55	85	9.29E-08	58.4	Low homology
			600182-1	100	31	0	0	175	205	33	3	9.29E-08	58.4	Low homology
			566744-1	100	31	0	0	175	205	39	9	9.29E-08	58.4	Low homology
			426341-1	100	31	0	0	175	205	92	62	9.29E-08	58.4	Low homology
			422449-1	100	31	0	0	175	205	92	62	9.29E-08	58.4	Low homology
			412670-1	100	31	0	0	175	205	82	52	9.29E-08	58.4	Low homology
			359558-1	100	31	0	0	175	205	33	3	9.29E-08	58.4	Low homology
			357275-1	100	31	0	0	175	205	62	32	9.29E-08	58.4	Low homology
			321875-1	100	31	0	0	175	205	48	18	9.29E-08	58.4	Low homology
			283343-1	100	31	0	0	175	205	48	18	9.29E-08	58.4	Low homology
			283226-1	100	31	0	0	175	205	37	7	9.29E-08	58.4	Low homology
			256022-1	100	31	0	0	175	205	44	14	9.29E-08	58.4	Low homology
			239164-2	100	31	0	0	175	205	88	58	9.29E-08	58.4	Low homology
			228440-2	100	31	0	0	175	205	31	1	9.29E-08	58.4	Low homology
			106416-3	100	31	0	0	175	205	35	5	9.29E-08	58.4	Low homology
			1352345-1	100	29	0	0	175	203	29	1	1.20E-06	54.7	Low homology
			1159973-1	100	29	0	0	177	205	118	90	1.20E-06	54.7	Low homology
			530191-1	100	29	0	0	177	205	53	25	1.20E-06	54.7	Low homology
			446459-1	100	29	0	0	175	203	29	1	1.20E-06	54.7	Low homology
			1347319-1	100	28	0	0	175	202	28	1	4.32E-06	52.8	Low homology
			1289412-1	100	28	0	0	175	202	28	1	4.32E-06	52.8	Low homology
			711297-1	100	28	0	0	175	202	56	29	4.32E-06	52.8	Low homology
			614048-1	100	28	0	0	175	202	28	1	4.32E-06	52.8	Low homology
18	EfM3.075550.mRNA-1	Hypothetical protein	921548-1	100	29	0	0	1106	1134	104	76	4.25E-06	54.7	Low homology
19	EfM3.079430.mRNA-1	Translin family protein	723944-1	100	49	0	0	44	92	3	51	3.81E-17	91.6	Highly conserved
			1162369-1	91.228	57	5	0	38	94	13	69	2.96E-13	78.7	Highly conserved
			1275315-1	92.453	53	4	0	38	90	13	65	1.07E-12	76.8	Highly conserved
			1271502-1	92.453	53	4	0	38	90	13	65	1.07E-12	76.8	Highly conserved
			1206387-1	92.453	53	4	0	38	90	13	65	1.07E-12	76.8	Highly conserved
			1206374-1	92.453	53	4	0	38	90	13	65	1.07E-12	76.8	Highly conserved
			1056684-1	92.453	53	4	0	38	90	13	65	1.07E-12	76.8	Highly conserved
			793501-1	92.453	53	4	0	38	90	13	65	1.07E-12	76.8	Highly conserved
			650669-1	92.453	53	4	0	38	90	13	65	1.07E-12	76.8	Highly conserved
			585435-1	95.745	47	2	0	44	90	19	65	1.07E-12	76.8	Highly conserved
			508225-1	92.453	53	4	0	38	90	13	65	1.07E-12	76.8	Highly conserved
			422522-1	92.453	53	4	0	38	90	13	65	1.07E-12	76.8	Highly conserved
			395373-1	95.745	47	2	0	44	90	19	65	1.07E-12	76.8	Highly conserved
			368756-1	92.453	53	4	0	38	90	13	65	1.07E-12	76.8	Highly conserved
			264395-1	92.453	53	4	0	38	90	13	65	1.07E-12	76.8	Highly conserved
			131772-3	92.453	53	4	0	38	90	13	65	1.07E-12	76.8	Highly conserved
			123911-3	92.453	53	4	0	38	90	13	65	1.07E-12	76.8	Highly conserved
			115061-3	92.453	53	4	0	38	90	13	65	1.07E-12	76.8	Highly conserved
			31056-10	92.453	53	4	0	38	90	13	65	1.07E-12	76.8	Highly conserved
			1167486-1	90.566	53	5	0	38	90	13	65	4.96E-11	71.3	Highly conserved
			1287701-1	92.5	40	3	0	38	77	13	52	3.86E-07	58.4	Low homology

Supplementary Information 4

(a)

```
LpFTRL          ATGGCAAG--------------CATGG--TCGT--GGGCGC--ATCATCCTTGCCTC-GA
E. festucae     ATGCCGAATCCATTCTGTCTCACCTGGCCTCGCCAGAGCGCGGAGAATTCCCCCCTCCGC
                *** * *               * ***  ***   * ****  *  ** *   **** *

LpFTRL          TGGGGTAGCTGCTCTCTCCATGGCCTCAACACTGAGGTCCGCA---CGGGGCTAGACAAA
E. festucae     TGGGGTGACTACTCTGTCCAAGGCCTCAACATTGAGGTCGTCGGGGCGGTGCCGGACAGG
                ******  ** **** **** ********** *******  *    *** **  ****

LpFTRL          GACGCTCTCCCAGGCGTTACCGGCGGCGGCATTAGAA-------TCCCTCCGTATTTCTG
E. festucae     GACGCTCTCCCCGGCGGCGACGGCAG-GCCATTTAAAATCATCATCTCTCCGTACTTCTG
                *********** ****    **** * * ****  **       ** ******* *****

LpFTRL          GGGCGAGGGTCCGGTGGCC-------------GAC---GCCG--------AGCTT-----
E. festucae     GGGCGAGGGTCCCGTGGCCAGGCTAGCGAGGCGACTTGGCTGGAAGGGGAAGCTGAGGAG
                ************ ******             ***   ** *        ****

LpFTRL          -------GGCTTCCCGGTGTACACAGGCTACTCGGAGGCGAGGGCCCTGATCGGCGAGGG
E. festucae     AGATGGAGGCTTCCCCGTGCACGCGGGCTACTCGGAGGCGAGGGCCTTGGTCGGCAAGGG
                       ******** *** ** * ********************* ** ***** ****

LpFTRL          AGCCACGGAGGATCTGGCAAGGCTGTCACGCCAGGCCCAGGACATGGTGGCCGAGATGTT
E. festucae     AGCCGTGACGGATCTGAGAAGGCTGTGCCTCGAGGCGGAAGATTTGGTGGAGGAGATGTT
                ****  *  *******  ********  * * ****  * **  ******  ********

LpFTRL          TGCTAGCATCGGCAGGGAC--------CAAGA----------------GGAAGCGG---C
E. festucae     TGCTAGCATCGGCGGCGGCGTCCGGCACAAGGACGAGCGGTTCGGGGCGGAAGCGGAGGT
                ************* * * *        ****                 ********

LpFTRL          GC-----GCGG----GCCCAGGGTGGTGCAGCTGCGGCTATCGCCGGAGCTCGCCCTGTG
E. festucae     GCCGGAAGCGGCGCGGCCCAGGGTGGTGCAGCTGCGGCTGTCGCCGGAGCTCGCGCTGTG
                **     ****    ************************ ************** *****
```

(a) (Cont'd)

```
LpFTRL        GAAAAGCATCCAACACGCAATCGGCAACGTGCTTCCCCCTAAGGGCAAGGGGCTGAAGCA
E. festucae   GAAAAGCACGCAGCACGCGATGGGCAAGGTGCTGCCCCCCAAGGGCGCGGGGCTGAGCCA
              ********  ** ***** ** ***** ***** ***** ******  ********  **

LpFTRL        AGCCTCACCGCAGGTGCTCGCCCAGCTGGGCGCCACCGACTGGCCCGAGGCCATCACCAC
E. festucae   AGCCTCGCCGCGGGTGCTCGCCGTGCTGGGGGCCGGCGACTGGCCCGAGGCCATGACGAC
              ****** **** **********  ****** ***  ****************** ** **

LpFTRL        CAACAATGCCCTGTTCGGCGGCGGCAGCACTGCCATGCTCATTGGCGCCGCCGACCTGCA
E. festucae   CACCAACGCCCTGTTCGGCTGCGGCATGGCGAGCCTGCTCATTGGCGCCGCCGACGTGCG
              ** *** ************ ******   *   * ******************** ***

LpFTRL        CACCATGTTCTCCAACTATGTGACCGACATGGCCGTTTACTACGAGTATGGGTACAACCA
E. festucae   CACCCTGTTCTCCAACTACGTGACCGACATGGCCTTTTACTACGAGCACGGGTACAACCA
              **** ************* *************** *********** * ***********

LpFTRL        TGTGTTCCCTCACCTCCACGGGATGCTGCAGGATGGGCTCGCCGACGCCCACGCGCTGGC
E. festucae   CGTCTTCCCCTCGTTCCACCGGCTGCTGCACGACGGGGTCGCCGACGCCCACGCGCGGCG
               ** *****     ***** ** ******* ** *** ****************** *

LpFTRL        CACCCCTTGCGGCCAGGAGCGCCGGGAGGCCGTCGCCGTAGGCCTCCCCTACATCCAGGG
E. festucae   CACCCTCGGCGGCCGGCAGCGGCGCGAGGCCGTCGCCGCCGGCCTGCGGTACATCCAGGC
              *****   ****** * **** ** *************  ***** *  **********

LpFTRL        CAAGATCGCGATGGAGGTGGCGGACAGAACACGTCTCAAGGACTTCTCCGCGCAGATGGA
E. festucae   CAAGATCGCGCTCGAGGCGGCGCACGGGACGCGCCTGAAGGACGCCGCCGCGCGGATGGA
              ********** * **** **** ** * ** ** ** ******  * ****** ******

LpFTRL        TCGTCGTGCCGCCCAGATCATTTTCCTCTTTGACTGCAGCGTGCTGGGCATTGGAGCCGA
E. festucae   CCGCCGGACCGCCCAGGTCATCTCCCTGTCCGAGAGCAGCCTGCTCGGCATGGCGGCCGA
               ** **  ******** **** * *** *  **  ***** **** ***** *  *****

LpFTRL        GGCCACGGCCCGGGGCTTCGACGCCGGCGCCGTCATGAGCGACCTCATCTTGAGTGTCAA
E. festucae   GGCCATGGCCCGGGGCTTCGACGCCGCCGCCGTCATGAGCGACCTCGTCTTCAGC-TCGC
              ***** ******************** ******************* **** **   **
```

(a) (Cont'd)

```
LpFTRL         CAGCCAC-GACGTCCTCGACGTGGGCTCCGACCTGGTCAACTCCGAGATCATGAACTCAT
E. festucae    CCGGCACCGACGTCGTCGACGTGGGCTGCGACCTGGTCAACTCCGAGGTCATGAACTCGT
               * * *** ****** ************ ******************* ********** *

LpFTRL         TCCTCAACGTCGCCGACATCGCCGCCTCGG------GCGTCGTGAGCGAGCCGGCGCTCC
E. festucae    TCCTCAACGTGGCCGACATCGCCGCCTCCGAGGGCGGCGTCGTGAGCGAGCCGGCGCTGC
               ********** ***************** *      ********************** *

LpFTRL         GGGCCATCTACGACGCATACGCTGCCACGGGCGCCCGGCTGTTCACCCAGAGGTGGCACG
E. festucae    GGGCCATCTACGACGCCTACGCCGCCACGGGCGCGCGCATGCTGACCCAGAGGTGGCACG
               **************** ***** *********** **  ** * ****************

LpFTRL         AGCCCTCGGCCCGGATGGTCGCCAATGAATACATCTGGCACATCGCCAACGACCGGCACA
E. festucae    AGCCCGTGGCCAGGATGTGCGCCGCGCTGTACACGTGGCACATCCAAAACGACCGGCACA
               *****  **** *****  ****      ****  *********   *************

LpFTRL         TGCTCTTCCGCCGCGCCCTCCTGGGATGGCCCATGGCCCGCAAGTCGCCGGCGTCACCCC
E. festucae    TGTTCCTCCGCCGCGCCCTCCTGGGATGGCCCAAGGCCCGCAAGTCGCCGGCGCGGCCCC
               ** ** *************************** *******************   ****

LpFTRL         AGCGTGAGGCCGACTTCGACGAGGTATTCGACGCCGACTTCCACACCACCGGCTTCAGCA
E. festucae    AGCGCGAGGCCGACTTCGACGAGGTCTTCGACGCCGACTTCCACACCACCGGCTTCAGCA
               **** ******************** **********************************

LpFTRL         GGCCCATCGACCCCGAATACGCCTGCGACGGCGAGGAAACCTGCAACCATGTGCGCCGGT
E. festucae    GGGCCCTCGGCCCCGCGTACGCCTGCGACGGCGGCGACACCTGCAACCACGTCCGTCGCT
               ** ** *** *****  ****************  ** *********** ** ** ** *

LpFTRL         TCCTCGACCGCC--------AGGA----TGAAGACCTGCTCAGCAGCTTCTGGTGGTCCC
E. festucae    TCCTCGACCGCCGCTCTCTGAGGGGTGGTGAGGACCTGCTCGGCGCCCTCTGGTCGTCTC
               ************        ***     *** ********* **  * ****** *** *

LpFTRL         TCGTCACCGGGCCGCTTGAGTACATCCGGCAGGGCGAGGTGGACGAGAAGCACGAGGAGC
E. festucae    TCGTCACCGGCCCGCTCGAGTACGTCCGGCGGGGCGAGGTGGACGAGCAGCGCGAGCAGC
               ********** ***** ****** ****** **************** *** **** ***
```

(a) (Cont'd)

```
LpFTRL           ACCTCATCGAATCCTCGCGCATGCAAATGGCCCAGCTCTTGTCCAAGGGCCTTGTCCTCG
E. festucae      ACCTCGCCGAATCCTCGCGCCTGCAAATGGCCCAGCTCTTCTCCAAGGGCCTCGTCGTCG
                 *****  ************* ******************* *********** *** ***

LpFTRL           AATTGACCTGGCTCCTCGCCCATGCGAGCCACCATGCCTGGCAGGTGAACTACATGTACG
E. festucae      AGATGGTCTGGCTGATTGCCCATGCGAGCCACCACGCCTGGCAGGTGAACTACCTGTTCG
                 *  **  ******  * ***************** ****************** *** **

LpFTRL           AGGCCGCCATGTTTGGAAGCATCTTGGATGGGGGCGCGTTGATAGGCAAGCTTGACCGGG
E. festucae      AGGCCGCCATGTTTGGCAGCATCCTGGACGGGGGCGCATTGATAGGCAAGCTCGACCGGG
                 **************** ****** **** ******** ************** *******

LpFTRL           ------------------------AAGAGAAGGG------------CTCCATCGGCT---
E. festucae      CAGAGGGCGAGGAAGCGCAAGACCAAGAGGAGGAGAAGAACATGACCTACATCAATTTCC
                                         ***** ***             ** ****   *

LpFTRL           -----GA-------------
E. festucae      GCAACGAACTGGGTTGTTAA
                      **
```

(b)

```
LpDUF3632   ------------------------------------------------------------
E. festucae AGTGAAGTCGCTTCTCCCTGCACAACCATGATTATATACATCTACCCTCTGCTCGAATGA

LpDUF3632   ------------------------------------------ATGCCGCCCGTCGTTGAG
E. festucae GGCCCAGTCGGTTGTTCCTCACCATTCATTCTCGCAATTACCATGCCGCCCGTTGTTCAG
                                                      *********** *** **

LpDUF3632   CTTCGCTTGGAATTTGCCTATGAAGGCGCGGTCTACTTCGCGCCGCGGGTCATCGAGATA
E. festucae CTTCGCTTGGAAGATGC------AGGTGCGCCCGACTTCGCGCCGCGGGTCATCGAGATA
            ************  ***      *** ***  * **************************

LpDUF3632   CTCAATTCGACGCTGCCCGTCG---ACGGCGCAAAGACGCCCGGCGAAGCCGCCGCCGCA
E. festucae CTCAATTCGACTCTGCCCGTCGTCAACGGTGCAAAGACGCCCGGCGAAGCCGCCGCGGCA
            *********** **********   **** ************************** ***

LpDUF3632   CTTGATGCCCTGTTCGATGAGAACTACTCCGCAGAAGCCGATGATAGCGCAGAGGGTTTT
E. festucae CTCGATGCCCTGTTCGGTGAGAACTAC---GTGGAAGCCGATG---GTGCAGGTGGATTC
            ** ************* **********   *  **********   * ****  ** **

LpDUF3632   CTGTGGTGGTTCTGGACCTTGATGCACGACCTGTCGCGCCAAGTGCCATACAACAGCCCG
E. festucae CTGTGGTGGTTCTGGGACTTGATGCACGATTTGTCGCGCCAAGTCCCATACAACAGCCCC
            ***************  ************  ************* ************** 

LpDUF3632   GAGGCTGAGAGGCTGGCGAGCACGCTTCAGGCGCTGCACGATCTGCCAACCAAATCCGTG
E. festucae GAGGCCGAGAGACTGGCAAGCACGCTTCAAGCGCTGCACGATCTTCCAACCAAATCTGTG
            ***** ***** ***** *********** ************** *********** ***

LpDUF3632   AAGCTCGGCAAGTCATGGGGCGGTGGAA---CCCTGGAGCAGTGGCGAGACATGCCCTTT
E. festucae AAACTCGGCGACTCTTGGGGTGCCGGAAGCACCGTGGAGCTGTGGCAAGACTTGCCCTTG
            ** ****** * ** ***** *  ****   ** ****** ***** **** ******* 

LpDUF3632   TTCGGCCCCACCTACCGCGAGGCCCTCGACTATGACCCCGGAGCAGCAGATGAAGAGGAC
E. festucae TTCGGCGCCACCTATCGCGAGGCCATCGACAATGACCCCAGAGCACCGAATGAGGAAGAA
            ****** ******* ********* ***** ******** ***** *  **** ** ** 
```

(b) (Cont'd)

```
LpDUF3632    AGGAAGCAGCGATTCGTGAACCTGCAGAGTTACGCGGCGAGGGTGGCAGGTCTGGGAGTC
E. festucae  AGGAAGCAGCGATTCGTGAACCTGCAGACTTACGCCGCGAGAGTGGCAGGTCTGGGACTC
             **************************** ****** ***** *************** **

LpDUF3632    ATCGAGGTGGATGGATGGTGCTTGTGGGCGCTGTGGACCCTGGTGGAGGCGCTCGAGGGC
E. festucae  ATCAAGGTGGACACATGGTCCTCGTGGGCGCTATGGACCCTGGTCGAAGCGCTCGAGGGC
             *** *******   ***** ** ********* *********** ** ************

LpDUF3632    TCCATGACGCCGGTCAGAGGTGCTCCGGATGAGATCAACGACGATCCAGCTGCTGTGCAG
E. festucae  TCCATGACGCCAATCAGGGGTGCTCCAGATGAGATCAACGAGGATCCAGCTGCTGTACAA
             ***********  **** ******** ************** ************** **

LpDUF3632    GGC------TACATGGTGAAGAGCGCCGCGGCATGGATCATCTTCGCCGGCAATCGTCTG
E. festucae  GACATCTCCTACAAGGTGAAGAGCGCCGCGGCATGGATCATCTTCGCGGGCAATCGTCTG
             * *      **** ********************************* ************

LpDUF3632    TATGGACGAGACGAGGAGGTCGTTGGTGCCTCAGCCGGTCCACTCTGGAGACTCAGCAAG
E. festucae  TATGGACGGGACGAGGAGGTCGTTGGTGCCACAGCCGGTCCACTCTGGAGACTCGACAAG
             ******** ********************* ***********************  ****

LpDUF3632    GAGGAAATAGTCAAGTTGAAGAGGAAGACGAAAGGAACAGATGGTTTTTGCCCGGAGCGC
E. festucae  AAGGAAGCAGTCAAGTTGAACAGGAAGACGAAGGGAACAGATGGTTATTGCCCGGAGCGC
              *****  ************ *********** ************* *************

LpDUF3632    TGGAATCTGTGGAAGCAGAGGTTCGCTCGGATAAGGGACGCGGACGAATTAGAGGCAGAT
E. festucae  TGGAATCTGTGGAAGCAGAGATTCGCTCGTATAAGGGACACGGACAAGTTGGAGGCAGAT
             ******************** ******** ********* ***** * ** *********

LpDUF3632    GTTCGGAACCAGGCGGGCTATGCGTTCGATGCAATGGAGGCGGCGGAGAAATGTCATACT
E. festucae  GTTCGGAACGAGGCGGGCTATGCGCTTACTGCAATGGAGGTGGTGGAGAAATTCCATACC
             ********* ************** *   *********** ** ********  *****

LpDUF3632    CCTACCTAG
E. festucae  TAG------
```

(c)

```
LpFTRL         -------MASMVVGASSLPRWGSCSLHGLNTEVR-TGLDKDALPGVTGG--GIRIPPYFW
E. festucae    MPNPFCLTWPRQSAENSPLRWGDYSVQGLNIEVVGAVPDRDALPGGDGRPFKIIISPYFW
                        .   . .*  ***. *::*** **  :  *:*****  *    * *.****

LpFTRL         GEGPVA------------DAELGFPVYTGYSEARALIGEGATEDLARLSRQAQDMVAEMF
E. festucae    GEGPVARLARRLGWKGKLRRDGGFPVHAGYSEARALVGKGAVTDLRRLCLEAEDLVEEMF
               ******              : ****::********:*:**. ** **. :*:*:* ***

LpFTRL         ASIG------------RDQEEAARGPRVVQLRLSPELALWKSIQHAIGNVLPPKGKGLKQ
E. festucae    ASIGGGVRHKDERFGAEAEVPEAARPRVVQLRLSPELALWKSTQHAMGKVLPPKGAGLSQ
               ****            . :   *  ***************** ***:*:****** **.*

LpFTRL         ASPQVLAQLGATDWPEAITTNNALFGGGSTAMLIGAADLHTMFSNYVTDMAVYYEYGYNH
E. festucae    ASPRVLAVLGAGDWPEAMTTTNALFGCGMASLLIGAADVRTLFSNYVTDMAFYYEHGYNH
               ***:*** *** *****:**.***** * :::******::*:*********.***:****

LpFTRL         VFPHLHGMLQDGLADAHALATPCGQERREAVAVGLPYIQGKIAMEVADRTRLKDFSAQMD
E. festucae    VFPSFHRLLHDGVADAHARRTLGGRQRREAVAAGLRYIQAKIALEAAHGTRLKDAAARMD
               *** :* :*:**:*****  *  *::******.** ***.***:*.*. ***** :*:**

LpFTRL         RRAAQIIFLFDCSVLGIGAEATARGFDAGAVMSDLILSVNSHDVLDVGSDLVNSEIMNSF
E. festucae    RRTAQVISLSESSLLGMAAEAMARGFDAAAVMSDLVFSSPGTDVVDVGCDLVNSEVMNSF
               **:**:* * :.*:**:.*** ******.******::*  . **:***.******:****

LpFTRL         LNVADIAAS--GVVSEPALRAIYDAYAATGARLFTQRWHEPSARMVANEYIWHIANDRHM
E. festucae    LNVADIAASEGGVVSEPALRAIYDAYAATGARMLTQRWHEPVARMCAALYTWHIQNDRHM
               *********  *********************::******* *** *  * *** *****

LpFTRL         LFRRALLGWPMARKSPASPQREADFDEVFDADFHTTGFSRPIDPEYACDGEETCNHVRRF
E. festucae    FLRRALLGWPKARKSPARPQREADFDEVFDADFHTTGFSRALGPAYACDGGDTCNHVRRF
               ::******** ****** **********************.:.* ***** :********
```

(c) (Cont'd)

```
LpFTRL          LDRQ----DEDLLSSFWWSLVTGPLEYIRQGEVDEKHEEHLIESSRMQMAQLLSKGLVLE
E. festucae     LDRRSLRGGEDLLGALWSSLVTGPLEYVRRGEVDEQREQHLAESSRLQMAQLFSKGLVVE
                ***:    .****.::* *********:*:*****::*:** ****:*****:*****:*

LpFTRL          LTWLLAHASHHAWQVNYMYEAAMFGSILDGGALIGKLDREEKGSIG--------------
E. festucae     MVWLIAHASHHAWQVNYLFEAAMFGSILDGGALIGKLDRAEGEEAQDQEEEKNMTYINFR
                :.**:************::******************** *   .

LpFTRL          -----
E. festucae     NELGC
```

(d)

```
LpDUF3632      MPPVVELRLEFAYEGAVYFAPRVIEILNSTLPVDG-AKTPGEAAAALDALFDENYSAEAD
E. festucae    MPPVVQLRLEDA--GAPDFAPRVIEILNSTLPVVNGAKTPGEAAAALDALFGENY--VEA
               *****:**** *  **  *************** . ***************.***

LpDUF3632      DSAEGFLWWFWTLMHDLSRQVPYNSPEAERLASTLQALHDLPTKSVKLGKSWGGG-TLEQ
E. festucae    DGAGGFLWWFWDLMHDLSRQVPYNSPEAERLASTLQALHDLPTKSVKLGDSWGAGSTVEL
               *.* ******* *************************************.***.* *:*

LpDUF3632      WRDMPFFGPTYREALDYDPGAADEEDRKQRFVNLQSYAARVAGLGVIEVDGWCLWALWTL
E. festucae    WQDLPLFGATYREAIDNDPRAPNEEERKQRFVNLQTYAARVAGLGLIKVDTWSSWALWTL
               *:*:*:**.*****:* ** *.:**:*********:*********:*:** *. ******

LpDUF3632      VEALEGSMTPVRGAPDEINDDPAAVQG--YMVKSAAAWIIFAGNRLYGRDEEVVGASAGP
E. festucae    VEALEGSMTPIRGAPDEINEDPAAVQDISYKVKSAAAWIIFAGNRLYGRDEEVVGATAGP
               **********:********:******.  * *************************:***

LpDUF3632      LWRLSKEEIVKLKRKTKGTDGFCPERWNLWKQRFARIRDADELEADVRNQAGYAFDAMEA
E. festucae    LWRLDKKEAVKLNRKTKGTDGYCPERWNLWKQRFARIRDTDKLEADVRNEAGYALTAMEV
               ****.*:* ***:********:*****************:*:*******:****: ***.

LpDUF3632      AEKCHTPT
E. festucae    VEKFHT--
               .** **
```

Supplementary Information 5

Common name	Scientific name	NCBI SRA UI	Tissues	Source	Instrument	Data size	*Lp* DUF3632		*Lp* FTRL		*Lp* BGNL		*Ef* MCF		Institute/Organisation
							Highest identit	Hit number	Highest identit	Hit number	Highest identit	Hit number	Highest identit	Hit number	
Italian ryegrass	*Lolium multiflorum*	SRX1604871	Whole plant	Transcriptome	Illumina HiSeq 2000	10.6G	100%, 3e-37	455	100%, 3e-37	124	100%, 3e-37	10905	N.S.		Department of Grassland Science, Sichuan Agricultural University
Tall fescue	*Festuca arundinacea*	SRX1056957	Leaves	Transcriptome	Illumina HiSeq 2000	5G	100%, 2e-37	100	94%, 2e-30	58	100%, 2e-37	914	N.S.		Chengdu Institute of Biology, Chinese Academy of Sciences
Orchard grass	*Dactylis glomerata*	ERX1842528	Leaves	Transcriptome	Illumina HiSeq 2000	11.1G	N.S.		90%, 9e-29	10	96%, 2e-37	5247	N.S.		Teagasc Crops Research Centre
Orchard grass	*Dactylis glomerata*	SRX738187	Leaves	Transcriptome	Illumina HiSeq 2000	32.6G	N.S.		92%, 5e-31	234	96%, 2e-36	8529	N.S.		Sichuan Agricultural University
Antarctic hairgrass	*Deschampsia antarctica*	SRX465632	Seedlings	Genome	Illumina HiSeq 2000	31G	N.S.		93%, 1e-32	12	N.S.		N.S.		Korea Polar Research Institute
Annual Poa	*Poa annua*	SRX745831	Leaf	Transcriptome	Illumina HiSeq 2000	25.9G	N.S.		97%, 4e-37	500	N.S.		N.S.		Auburn University
Supina bluegrass	*Poa supina*	SRX745855	Leaf	Transcriptome	Illumina HiSeq 2000	8.9G	80%, 8.5*	1*	94%, 2e-34	103	N.S.		N.S.		Auburn University
Early meadow-grass	*Poa infirma*	SRX745858	Leaf	Transcriptome	Illumina HiSeq 2000	9.3G	N.S.		82%, 2e-16**	1**	N.S.		N.S.		Auburn University
Harding grass	*Phalaris aquatica*	SRX669405	Leaf	Transcriptome	Illumina HiSeq 2000	10.2G	N.S.		N.S.**		N.S.		N.S.		Teagasc

Supplementary Information 6

(a)

*Lp*DUF3632 primers

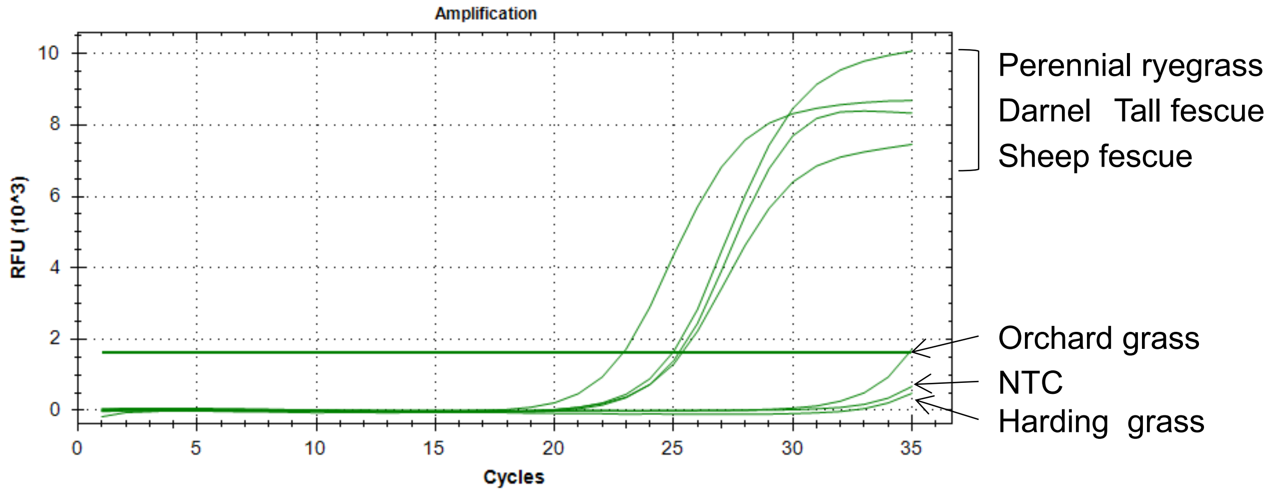

*Lp*FTRL primers

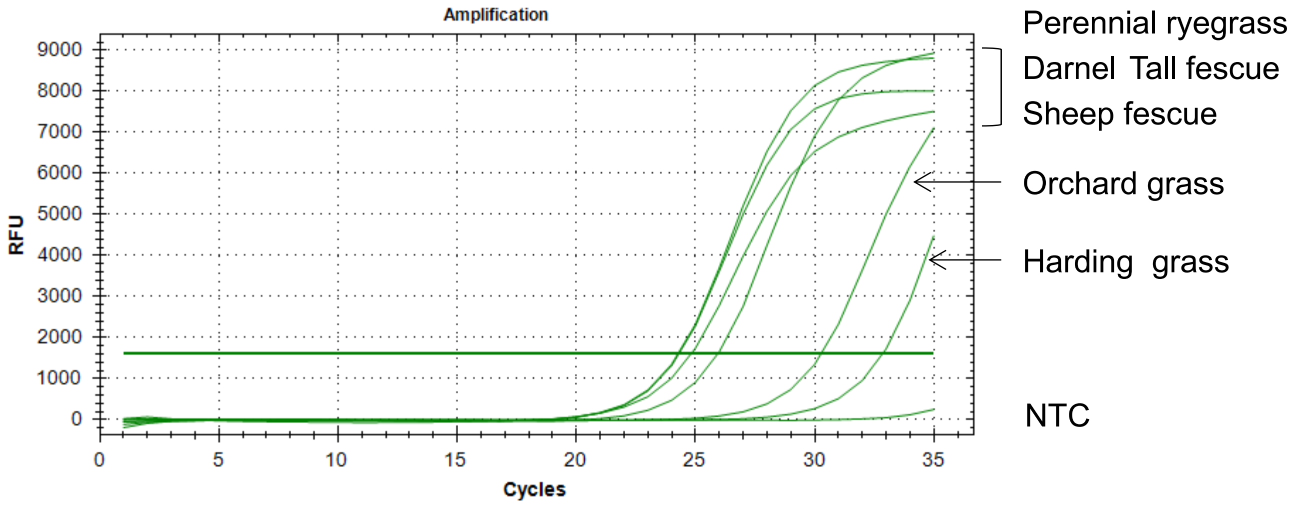

(b)

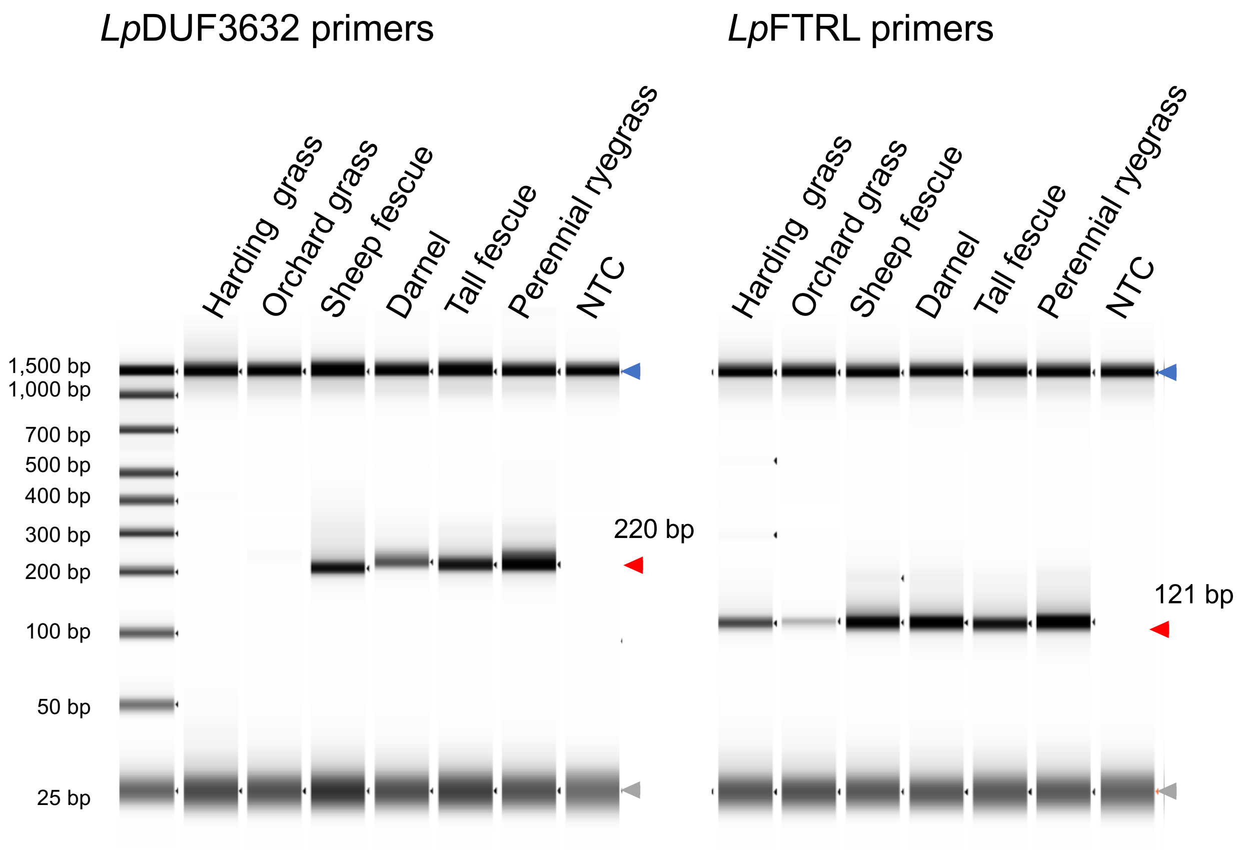
LpDUF3632 primers
LpFTRL primers
Harding grass
Orchard grass
Sheep fescue
Darnel
Tall fescue
Perennial ryegrass
NTC
1,500 bp
1,000 bp
700 bp
500 bp
400 bp
300 bp
200 bp
100 bp
50 bp
25 bp
220 bp
121 bp

Supplementary Information 7

(a)

```
C3_hap1         GTCGTTGAGCTTCGCTTGGAATTTGACTATGGAGGCGCGGTCTACTTCGCGCCGCGGGTC
C3_hap2         GTCGTTGAGCTTCGCTTGGAATTTGCCTATGAAGGCGCGGTCTACTTCGCGCCACGGGTC
                ************************* ***** ********************* ******

C3_hap1         ATCGAGATACTCAATTCGACGCTGCCCGTCGACGGCGCAAAGACGCCCGTCAAAGCCGCC
C3_hap2         ATCGAGATACTCAATTCGACGCTGCCCGTCGACGGCGCAAAGACGCCCGTCGAAGCCGCC
                *************************************************** ********

C3_hap1         GCCGCACTTGATGCCCTGTTCGGCGAGAACTACTCCGCAGAAGCCGATGATAGCGCAGAG
C3_hap2         GCCGCACTTGATGCCCTGTTCGATGAGAACTACTCCGCAGAAGCCGATGATAGCGCAGAG
                **********************  ************************************

C3_hap1         GGTTTTCTGTGGTGGTTCTGGGCCCTGATGCACGACCTGTCGCGCCAAGTGCCATACAAC
C3_hap2         GGTTTTCTGTGGTGGTTCTGGGCCTTGATGCACGACCTGTCGCGCCAAGTGCCACACAAC
                ************************ ***************************** *****

C3_hap1         AGCCCGGAGGCCGAGAGGCTGGCAAGCACGCT
C3_hap2         AGCCCGGAGGCTGAGAGGCTGGCAAGCACGCT
                *********** ********************
```

(b)

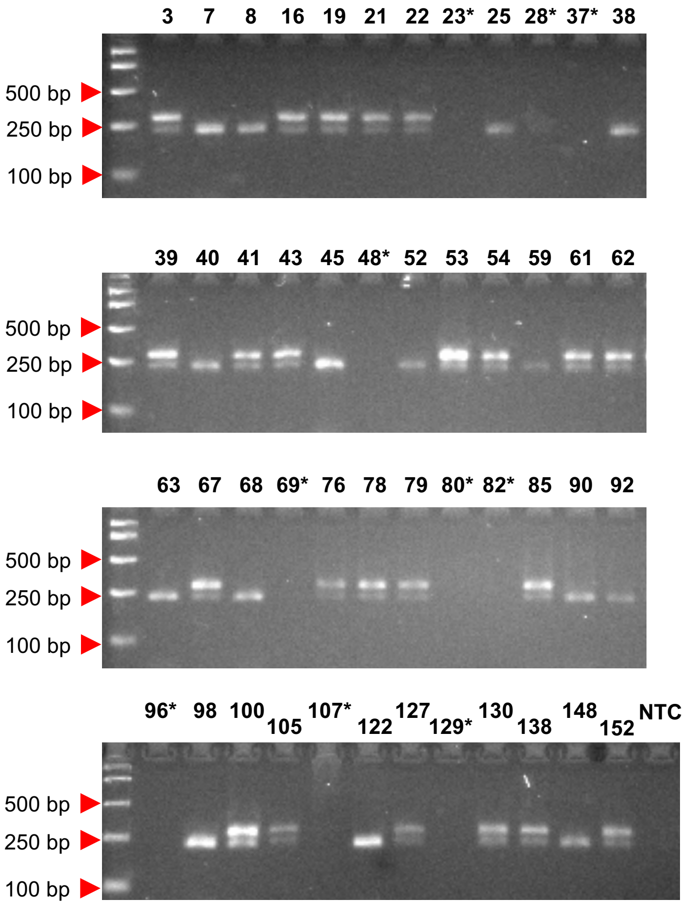
3 7 8 16 19 21 22 23* 25 28* 37* 38
500 bp
250 bp
100 bp
39 40 41 43 45 48* 52 53 54 59 61 62
500 bp
250 bp
100 bp
63 67 68 69* 76 78 79 80* 82* 85 90 92
500 bp
250 bp
100 bp
96* 98 100 105 107* 122 127 129* 130 138 148 152 NTC
500 bp
250 bp
100 bp

Supplementary Information 8

Tissues	NCBI SRA UI	Total read number	*Lp* FTRL		*Lp* DUF3632	
			Counts	CPM	Counts	CPM
Root tip	SRX2682771	19,599,475	13	0.66	217	11.07
Root (middle)	SRX2682772	19,345,991	6	0.31	145	7.50
Leaf middle 1	SRX2682778	31,634,846	1	0.03	139	4.39
Leaf middle 2	SRX2682777	36,003,206	0	0	151	4.19
Leaf middle 3	SRX2682776	34,518,849	0	0	104	3.01
Leaf tip 1	SRX2682781	36,794,815	2	0.05	198	5.38
Leaf tip 2	SRX2682780	37,693,192	195	5.17	138	3.66
Leaf tip 3	SRX2682779	30,485,271	135	4.43	82	2.69
Flower	SRX2682770	15,539,870	1	0.06	54	3.47

Supplementary Information 9

Endophyte status	Tissues	SRA UI	Total read number	*Lp* FTRL		EfM3.066060		*Lp* DUF3632		EfM3.028800	
				Counts	CPM	Counts	CPM	Counts	CPM	Counts	CPM
E^-	Root	SRX1167583	137,753,230	0	0	0	0	235	1.71	0	0
	Lower emerging leaf and stem	SRX1167581	95,765,204	0	0	0	0	99	1.03	0	0
	Sheath1	SRX1167585	100,523,270	0	0	0	0	458	4.56	0	0
	Sheath2	SRX1167587	97,147,346	0	0	0	0	460	4.74	0	0
	Blade 1	SRX1167577	78,951,230	0	0	0	0	133	1.68	0	0
	Blade 2	SRX1167579	166,142,848	0	0	0	0	357	2.15	0	0
	Upper emerging leaf	SRX1167589	102,264,294	0	0	0	0	194	1.90	0	0
E^+	Root	SRX1167584	136,231,854	0	0	0	0	273	2.00	2	0.01
	Lower emerging leaf and stem	SRX1167582	280,544,494	0	0	0	0	353	1.26	14	0.05
	Sheath1	SRX1167586	207,336,476	0	0	4	0.02	1,063	5.13	415	2.00
	Sheath2	SRX1167588	174,091,282	0	0	0	0	753	4.33	153	0.88
	Blade 1	SRX1167578	113,758,512	0	0	0	0	199	1.75	17	0.15
	Blade 2	SRX1167580	159,461,288	1	0.01	0	0	477	2.99	20	0.13
	Upper emerging leaf	SRX1167590	284,950,104	0	0	0	0	602	2.11	14	0.05

Supplementary Information 10

			Lp FTRL		EfM3.066060		*Lp* DUF3632		EfM3.028800	
Endophyte status	Time	Total read number	Counts	CPM	Counts	CPM	Counts	CPM	Counts	CPM
E^-	0 h	54,109,844	64	1.18	N.A.		383	7.08	N.A.	
	4 h	80,940,429	55	0.68	N.A.		801	9.90	N.A.	
	1 day	74,816,807	0	0	N.A.		773	10.33	N.A.	
	2 days	71,777,588	75	1.04	N.A.		520	7.24	N.A.	
	5 days	59,158,092	40	0.68	N.A.		439	7.42	N.A.	
	10 days	102,082,950	147	1.44	N.A.		887	8.69	N.A.	
E^+	0 h	36,385,117	7	0.19	18	0.49	157	4.31	8	0.22
	4 h	42,012,070	8	0.19	7	0.17	1234	29.37	13	0.31
	1 day	60,055,920	6	0.10	41	0.68	1014	16.88	6	0.10
	2 days	57,469,591	5	0.09	27	0.47	377	6.56	29	0.50
	5 days	57,257,627	41	0.72	6	0.10	374	6.53	10	0.17
	10 days	53,748,829	35	0.65	11	0.20	398	7.40	19	0.35

Supplementary Information 11

(a)

>LpDUF3632(ORF)

ATGCCGCCCGTCGTTGAGCTTCGCTTGGAATTTGCCTATGAAGGCGCGGTCTACTTCGCGCCGCGGGTCATCGAGATACTCAATTCGACGCTGCCCGTCG---
ACGGCGCAAAGACGCCCGGCGAAGCCGCCGCCGCACTTGATGCCCTGTTCGATGAGAACTACTCCGCAGAAGCCGATGATAGCGCAGAGGGTTTTCTGTGGTGGTTCTGGA
CCTTGATGCACGACCTGTCGCGCCAAGTGCCATACAACAGCCCGGAGGCTGAGAGGCTGGCGAGCACGCTTCAGGCGCTGCACGATCTGCCAACCAAATCCGTGAAGCTCG
GCAAGTCATGGGGCGGTGGAA---
CCCTGGAGCAGTGGCGAGACATGCCCTTTTTCGGCCCCACCTACCGCGAGGCCCTCGACTATGACCCCGGAGCAGCAGATGAAGAGGACAGGAAGCAGCGATTCGTGAACC
TGCAGAGTTACGCGGCGAGGGTGGCAGGTCTGGGAGTCATCGAGGTGGATGGATGGTGCTTGTGGGCGCTGTGGACCCTGGTGGAGGCGCTCGAGGGCTCCATGACGCCG
GTCAGAGGTGCTCCGGATGAGATCAACGACGATCCAGCTGCTGTGCAGGGC------
TACATGGTGAAGAGCGCCGCGGCATGGATCATCTTCGCCGGCAATCGTCTGTATGGACGAGACGAGGAGGTCGTTGGTGCCTCAGCCGGTCCACTCTGGAGACTCAGCAAG
GAGGAAATAGTCAAGTTGAAGAGGAAGACGAAAGGAACAGATGGTTTTTGCCCGGAGCGCTGGAATCTGTGGAAGCAGAGGTTCGCTCGGATAAGGACGCGGACGAATT
AGAGGCAGATGTTCGGAACCAGGCGGGCTATGCGTTCGATGCAATGGAGGCGGCGGAGAAATGTCATACTCCTACCTAG

>Epichlo_festucae_E2368_EfM3.028800.mRNA-1_(computed_transcript)
AGTGAAGTCGCTTCTCCCTGCACAACCATGATTATATACATCTACCCTCTGCTCGAATGAGGCCCAGTCGGTTGTTCCTCACCATTCATTCTCGCAATTACCATGCCGCCCGTTG
TTCAGCTTCGCTTGGAAGATGC------
AGGTGCGCCCGACTTCGCGCCGCGGGTCATCGAGATACTCAATTCGACTCTGCCCGTCGTCAACGGTGCAAAGACGCCCGGCGAAGCCGCCGCGGCACTCGATGCCCTGTTC
GGTGAGAACTAC---GTGGAAGCCGATG---
GTGCAGGTGGATTCCTGTGGTGGTTCTGGGACTTGATGCACGATTTGTCGCGCCAAGTCCCATACAACAGCCCCGAGGCCGAGAGACTGGCAAGCACGCTTCAAGCGCTGCA
CGATCTTCCAACCAAATCTGTGAAACTCGGCGACTCTTGGGGTGCCGGAAGCACCGTGGAGCTGTGGCAAGACTTGCCCTTGTTCGGCGCCACCTATCGCGAGGCCATCGAC
AATGACCCCAGAGCACCGAATGAGGAAGAAAGGAAGCAGCGATTCGTGAACCTGCAGACTTACGCCGCGAGAGTGGCAGGTCTGGGACTCATCAAGGTGGACACATGGTC
CTCGTGGGCGCTATGGACCCTGGTCGAAGCGCTCGAGGGCTCCATGACGCCAATCAGGGGTGCTCCAGATGAGATCAACGAGGATCCAGCTGCTGTACAAGACATCTCCTAC
AAGGTGAAGAGCGCCGCGGCATGGATCATCTTCGCGGGCAATCGTCTGTATGGACGGGACGAGGAGGTCGTTGGTGCCACAGCCGGTCCACTCTGGAGACTCGACAAGAA
GGAAGCAGTCAAGTTGAACAGGAAGACGAAGGGAACAGATGGTTATTGCCCGGAGCGCTGGAATCTGTGGAAGCAGAGATTCGCTCGTATAAGGGACACGGACAAGTTGG
AGGCAGATGTTCGGAACGAGGCGGGCTATGCGCTTACTGCAATGGAGGTGGTGGAGAAATTCCATACCTAG------

(b)

Synonymous substitution (Ks)	0.2725
Non-synonymous substitution (Ka)	0.0861
Ka/Ks	0.3160

Supplementary Information 12

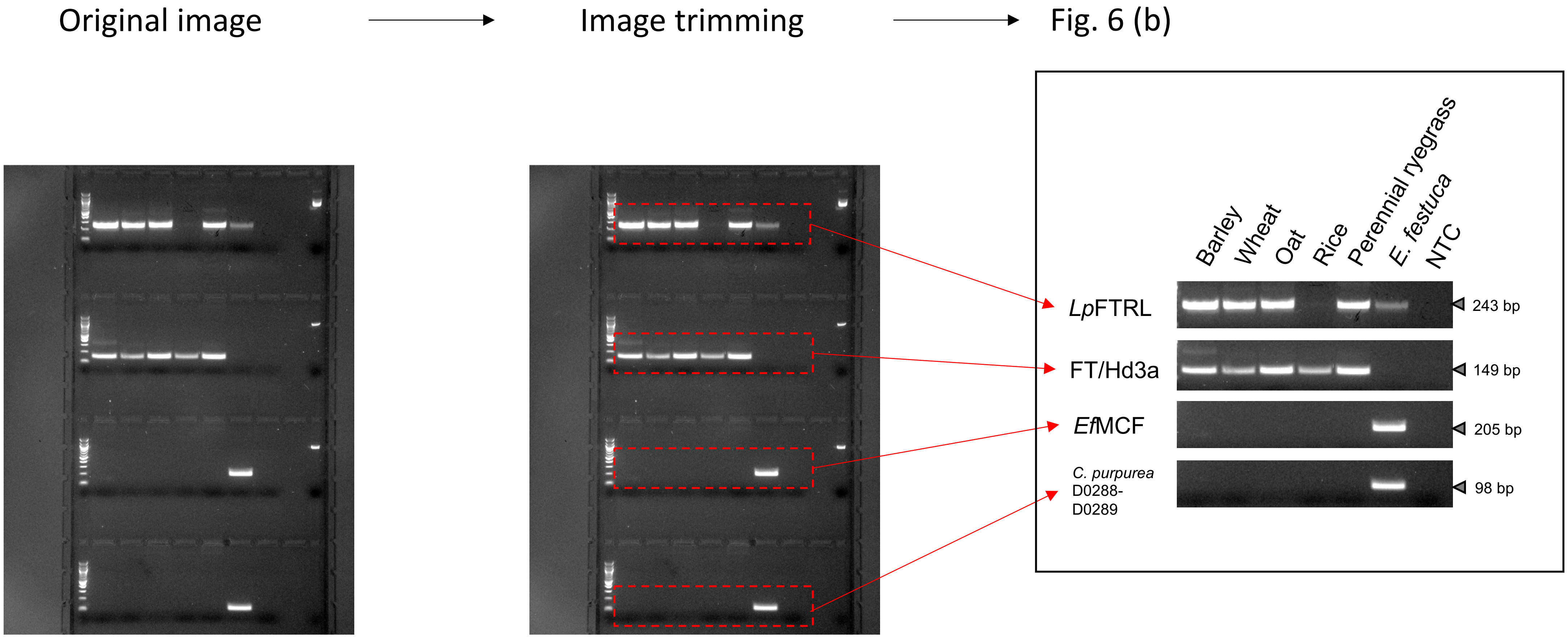

Supplementary Information 13

Species specificity	Target gene	Primer name	Sequence (5'->3')	Amplicon size
Plants	FTRL	LpFTRL_F	TCGACGTGGGCTCCGACCT	121 bp**
		LpFTRL_R	GCGTATGCGTCGTAGATGG	
Plants	DUF3632	LpDUF3632_F	CGCGGGTCATCGAGATACTC	220 bp**
		LpDUF3632_R	AGCGTGCTYGCCAGYCTCTC	
Plants/fungi	FTRL	FTRL_P-F_con_F	GGCCATCTACGACGCMTACG	243 bp**
		FTRL_P-F_con_R	GGCCTGCTGAAGCCGGTGGT	
Plants	FT/Hd3a	FT-HD3_P_con_F	GAGGTGATGTGCTACGAGAG	149 bp**
		FT-HD3_P_con_R	AGGTTGTAGAGCTCGGCRAA	
Fungi	mcf(*Ef* MCF)	Epichloe_mcf_F	TCGGATCATAGAAATGCCAC	205 bp***
		Epichloe_mcf_R	AAGCGATGTTTGGAAGATCT	
Fungi	ITS*	C.purpurea_D0288F	AGCCTTCTTTGCGTAGTA	98 bp*
		C.purpurea_D0289R	ACCTGATTCGAGGTCAAC	
Plants	FTRL	LpFTRL_SCA_F	CTCGTCCACCTCGCCCTG	1226 bp**
		LpFTRL_SCA_R	GCTACTCGGAGGCGAGGG	
Plants	DUF3632	LpDUF3632_SCA_F	GTCGTTGAGCTTCGCTTGGA	909 bp**
		LpDUF3632_SCA_R	ATCCGAGCGAACCTCTGCTT	

*Reference: 15. Comte, A. *et. al*. (2017) PLOS ONE 12, e0173495

**Amplicon size based on the perennial ryegrass genome sequence

***Amplicon size based on the *Epichloe poae* isolate Ps1 makes caterpillars floppy protein gene (GenBank UI: KJ502561.1)

Supplementary Information 14

(a) *Lp*FTRL

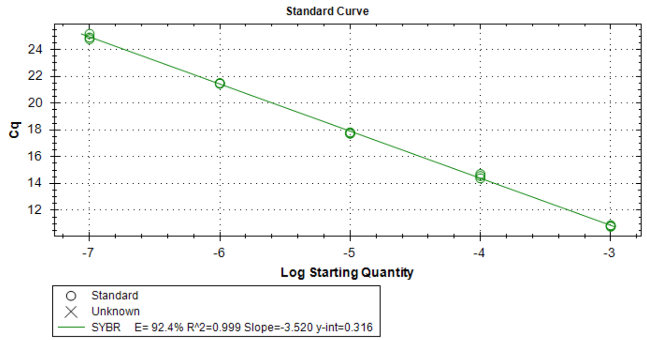

(c) *Ef*mcf

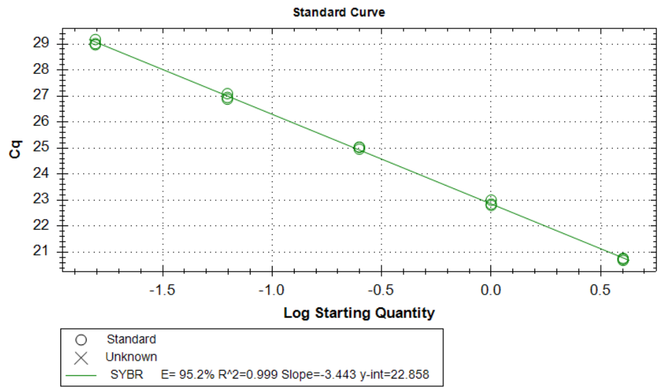

(b) *Lp*DUF3632

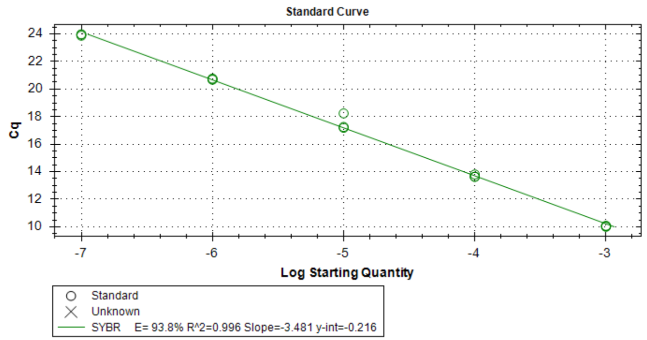

(d) *FTRL* (plants-fungi conserved)

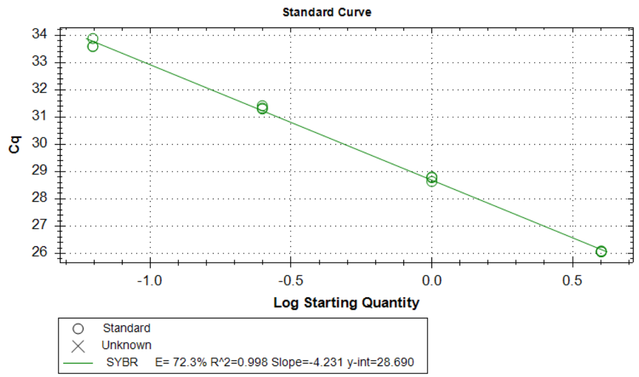

Supplementary Information 15

(supplied as a separate txt file)

Supplementary Information 16

(supplied as a separate txt file)

Supplementary Information 17

(supplied as a separate Word file)
