# Supplementary material for: Fungus-originated genes in the genomes of cereal and pasture grasses acquired through ancient lateral transfer
Source: Sci Rep. 2020 Nov 16;10:19883. doi: 10.1038/s41598-020-76478-4 (PMC7670438; doi:10.1038/s41598-020-76478-4)
Supplement: Supplementary file 6 — Supplementary Information 5. [file 41598_2020_76478_MOESM5_ESM.docx]

1. **Figure legend for the phylogeny including *Lp*FTRL**

Figure. Molecular Phylogenetic analysis by Maximum Likelihood method

The evolutionary history was inferred by using the Maximum Likelihood method based on the JTT matrix-based model [1]. The tree with the highest log likelihood (-8654.23) is shown. The percentage of trees in which the associated taxa clustered together is shown next to the branches. Initial tree(s) for the heuristic search were obtained automatically by applying Neighbor-Join and BioNJ algorithms to a matrix of pairwise distances estimated using a JTT model, and then selecting the topology with superior log likelihood value. The tree is drawn to scale, with branch lengths measured in the number of substitutions per site. The analysis involved 20 amino acid sequences. There were a total of 753 positions in the final dataset. Evolutionary analyses were conducted in MEGA7 [2].

1. Jones D.T., Taylor W.R., and Thornton J.M. (1992). The rapid generation of mutation data matrices from protein sequences. Computer Applications in the Biosciences 8: 275-282.

2. Kumar S., Stecher G., and Tamura K. (2016). MEGA7: Molecular Evolutionary Genetics Analysis version 7.0 for bigger datasets.Molecular Biology and Evolution 33:1870-1874.

Disclaimer: Although utmost care has been taken to ensure the correctness of the caption, the caption text is provided "as is" without any warranty of any kind. Authors advise the user to carefully check the caption prior to its use for any purpose and report any errors or problems to the authors immediately (www.megasoftware.net). In no event shall the authors and their employers be liable for any damages, including but not limited to special, consequential, or other damages. Authors specifically disclaim all other warranties expressed or implied, including but not limited to the determination of suitability of this caption text for a specific purpose, use, or application.

1. **Figure legend for the phylogeny including *Lp*DUF3632**

Figure. Molecular Phylogenetic analysis by Maximum Likelihood method

The evolutionary history was inferred by using the Maximum Likelihood method based on the JTT matrix-based model [1]. The tree with the highest log likelihood (-2582.22) is shown. Initial tree(s) for the heuristic search were obtained automatically by applying Neighbor-Join and BioNJ algorithms to a matrix of pairwise distances estimated using a JTT model, and then selecting the topology with superior log likelihood value. The tree is drawn to scale, with branch lengths measured in the number of substitutions per site. The analysis involved 24 amino acid sequences. All positions containing gaps and missing data were eliminated. There were a total of 205 positions in the final dataset. Evolutionary analyses were conducted in MEGA7 [2].

1. Jones D.T., Taylor W.R., and Thornton J.M. (1992). The rapid generation of mutation data matrices from protein sequences. Computer Applications in the Biosciences 8: 275-282.

2. Kumar S., Stecher G., and Tamura K. (2016). MEGA7: Molecular Evolutionary Genetics Analysis version 7.0 for bigger datasets.Molecular Biology and Evolution 33:1870-1874.

Disclaimer: Although utmost care has been taken to ensure the correctness of the caption, the caption text is provided "as is" without any warranty of any kind. Authors advise the user to carefully check the caption prior to its use for any purpose and report any errors or problems to the authors immediately (www.megasoftware.net). In no event shall the authors and their employers be liable for any damages, including but not limited to special, consequential, or other damages. Authors specifically disclaim all other warranties expressed or implied, including but not limited to the determination of suitability of this caption text for a specific purpose, use, or application.
